# Unraveling the Impact of Graphene Addition to Thermoelectric SrTiO_3_ and La-Doped SrTiO_3_ Materials: A Density Functional Theory Study

**DOI:** 10.1021/acsami.1c10865

**Published:** 2021-08-18

**Authors:** Joshua Tse, Alex Aziz, Joseph M. Flitcroft, Jonathan M. Skelton, Lisa J. Gillie, Stephen C. Parker, David J. Cooke, Marco Molinari

**Affiliations:** † Department of Chemical Sciences, University of Huddersfield, Queensgate, Huddersfield HD1 3DH, U.K.; ‡ Department of Chemistry, Queen Mary University of London, Mile End Road, London E1 4NS, U.K.; § Department of Chemistry, University of Manchester, Oxford Road, Manchester M13 9PL, U.K.; ∥ Department of Chemistry, University of Bath, Bath BA2 7AY, U.K.

**Keywords:** thermoelectrics, graphene/strontium titanate composite materials, electronic structure, structural dynamics, thermal transport, graphene adsorption on perovskite oxides

## Abstract

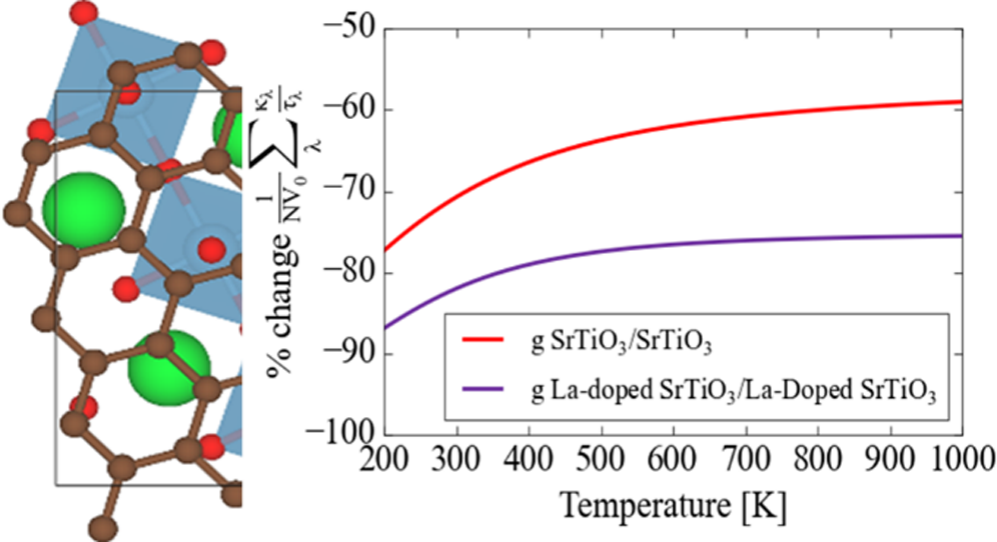

We present a detailed theoretical investigation of the interaction of graphene with the SrO-terminated (001) surface of pristine and La-doped SrTiO_3_. The adsorption of graphene is thermodynamically favorable with interfacial adsorption energies of −0.08 and −0.32 J/m^2^ to pristine SrTiO_3_ and La-doped SrTiO_3_ surfaces, respectively. We find that graphene introduces C 2p states at the Fermi level, rendering the composite semimetallic, and thus the electrical properties are predicted to be highly sensitive to the amount and quality of the graphene. An investigation of the lattice dynamics predicts that graphene adsorption may lead to a 60–90% reduction in the thermal conductivity due to a reduction in the phonon group velocities, accounting for the reduced thermal conductivity of the composite materials observed experimentally. This effect is enhanced by La doping. We also find evidence that both La dopant ions and adsorbed graphene introduce low-frequency modes that may scatter heat-carrying acoustic phonons, and that, if present, these effects likely arise from stronger phonon–phonon interactions.

## Introduction

With continued growth in global energy consumption, greater importance must be placed on moving away from fossil fuels to clean energy solutions. An increase in renewable energy generation must also be delivered alongside technologies to improve the efficiency of existing energy-intensive systems such as internal combustion engines to mitigate the worst effects of climate change.^1^ Thermoelectric (TE) power generation is a promising method to reduce energy consumption and increase efficiencies by recycling waste heat back to electrical energy.^2^ TE devices have already found applications in the aerospace, automotive and heavy manufacturing industries, and in remote power generation, and further improvements to their performance would allow them to compete economically with traditional sources of primary energy generation.^3−5^

The performance of a TE material is determined by the dimensionless figure of merit *ZT* = *S*^2^σ*T*/κ, where *S* is the Seebeck coefficient, σ is the electrical conductivity, κ *=* κ_el_ + κ_latt_ is the thermal conductivity, and *T* is the temperature. The thermal conductivity has contributions from the electrical thermal conductivity κ_el_ and the lattice (phonon) thermal conductivity κ_latt_. Methods for optimizing *ZT* include doping and dimensionality reduction^6−9^ through nanostructuring^10−12^ and nanocompositing.^8,13,14^ However, improving TE performance is a complex process due to the interdependence of the electrical properties—increasing σ by increasing the carrier concentration *n* improves the power factor *S*^2^σ but also increases κ_el_ and tends to decrease *S*.

Most TE materials currently on the market are alloys containing bismuth, antimony, and tellurium,^15−18^ which are rare, toxic, and/or expensive. While the highest *ZT* are currently obtained with chalcogenide TEs,^19,20^ recent work has shown that metal oxides^20−22^ such as CaMnO_3_,^23^ SrTiO_3_,^24−27^ M_2_CoO_3_,^28^ BaTiO_3_,^29^ tungsten bronze,^30,31^ Bi_2_Sr_2_Co_1.8_O*_y_*,^32^ In_2_O_3_,^33^ and La_1/3_NbO_3_^34^ may be promising alternatives to conventional TE materials while being made from cheaper, more earth-abundant, and less toxic materials.^35,36^ Among the potential oxide TEs, strontium titanate (SrTiO_3_; STO) shows promise due to its thermal stability at high temperature and tolerance to doping.^26,27,37^ Bulk SrTiO_3_ has a relatively poor *ZT*, and thus doping has been extensively explored as a possible route to improving its performance with examples including substituting Sr sites with rare-earth elements such as La^38,39^ and Ti sites with other transition metals such s Nb.^40−42^ La doping has been shown to promote the formation of A-site vacancies, which both increases the electrical conductivity and decreases the thermal conductivity through enhanced phonon scattering.^43^ Doping with La under reducing conditions further leads to the formation of oxygen vacancies, which decreases the thermal conductivity while having minimal impact on the electrical conductivity. Wang *et al*.^38^ showed that doping SrTiO_3_ with 12% Dy and 8% La increased the *ZT* from 0.05 to 0.36 at ∼800 °C.

As an alternative to doping, strain engineering,^44,45^ grain boundary engineering,^10,11,46,47^ and nanocompositing SrTiO_3_ with materials such as graphene also have the potential to improve the TE performance. In the latter case, whereas a graphene-based TE would perform poorly due to its large thermal conductivity, the addition of graphene to conventional-^48,49^ and oxide-based^8,13,14,50,51^ TEs has been shown to produce synergistic improvements in the *ZT*. Lin *et al*.^13^ investigated the addition of graphene to lanthanum-doped (LSTO) and noted a widening of the operating window to room temperature, a decrease in the thermal conductivity, and a significant increase in the electrical conductivity. The best results were obtained when 0.6 wt % of graphene was added, achieving a *ZT* of 0.42 at room temperature and 0.36 at 750 °C, amounting to a 280% improvement over pure LSTO. Bantawal *et al*.^50^ and Baran *et al*.^52^ investigated the electronic structure of graphene–SrTiO_3_ composites and found that carbon states were present at the Fermi level within the SrTiO_3_ band gap. The latter study^52^ also showed that by altering the SrTiO_3_ surface termination the electrical properties of the material could be tuned to optimize the electrical conductivity and to obtain both p and n-type transport.

In this work, we extend these previous studies by performing further detailed characterization of STO/LSTO–graphene composites, including modeling the structural dynamics and elucidating the impact of graphene adsorption on the thermal transport. We find that the nanocompositing produces surface interactions that impact both the electronic and thermal properties of the composite. Analysis of the electronic density of states displays characteristic fingerprints of graphene, which are reflected in the electrical properties of the composite, and analysis of the phonon spectra and group velocities indicates that the La doping and graphene adsorption are both likely to suppress the heat transport in the composite materials. These results provide further fundamental insight into how surfaces and interfaces can be exploited through nanocompositing, providing a firm foundation for the discovery and optimization of future high-performance TEs.

## Methodology

Calculations were performed using pseudopotential plane-wave density functional theory (DFT) as implemented in the Vienna *Abinitio* Simulation Package (VASP) code.^53−56^ We employed the Perdew–Burke–Ernzerhof (PBE) generalized-gradient approximation (GGA) functional^57,58^ with the DFT-D3 dispersion correction^59^ to account for van der Waals interactions. The projector augmented-wave (PAW) method was used to describe interactions between the core and valence electrons, with the following valence configurations: O—2s^2^2p^4^; C—2s^2^2p^2^; Sr—4s^2^4p^6^5s^2^, Ti—3d^2^4s^2^, and La—5s^2^5p^6^6s^2^5d^1^.^60,61^ A kinetic energy cutoff of 520 eV was used to represent the Kohn–Sham orbitals, which was determined from explicit convergence testing. Γ-centered ***k***-point grids with 6 × 6 × 6 and 4 × 4 × 1 subdivisions were used to integrate the Brillouin zones of bulk SrTiO_3_ and surface models, respectively. Surface models were generated with an initial vacuum spacing of 20 Å between periodic images, and a dipole correction was applied in the direction of the surface normal. The electronic energies were converged to 10^–6^ eV, and geometry optimizations were performed until the magnitude of the forces on the atoms were below 10^–2^ eV/Å. For the surface models, geometry optimizations were performed with the cell shapes and volumes fixed.

Transport coefficients were evaluated by solving the linearized Boltzmann transport equations within the constant relaxation-time approximation using the BoltzTraP code.^62^ The band energies of bulk SrTiO_3_ and the surface models were calculated non-self-consistently on dense 50 × 50 × 50 and 20 × 20 × 1 ***k***-point meshes, respectively, and Fourier interpolation was used to increase these densities by a further 5×. The BoltzTraP results were compared to calculations using the BoltzWann code,^63,64^ details of which are given in the SI. To obtain the charge carrier concentration, we used Boltzmann transport analysis, where the chemical potential is directly related to the carrier concentration. As the chemical potential is shifted into the valence or conduction bands, the number of hole or electron carriers increases. By interpolating the density of states (DOS), the number of carriers at any given chemical potential can thus be determined. To validate the BoltzTrap and BoltzWann methods, we calculated the Seebeck coefficient of bulk SrTiO_3_ as a function of carrier concentration (Figure S5) and as a function of temperature at a fixed carrier concentration corresponding to the bottom of the conduction band (Figure S6).^62,65^ The calculations with the two methods give similar results (Figures S7 and S8). We also compared the band structures of bulk SrTiO_3_ and the surface models obtained from our DFT calculations and by Wannier interpolation using the Wannier functions employed in our transport calculations (Figures S2–S4).

Finally, we employed the Phonopy^66^ and Phono3py codes^68^ to compute for the slab models the harmonic force constants, phonon spectra, phonon group velocities, and quantities related to the lattice thermal conductivity. In these calculations, VASP was used as the force calculator to obtain the harmonic force constants using the supercell finite-displacement method.^67^ Atomic displacements were generated in 2 × 2 × 1 supercells, and accurate single-point force calculations were performed with a 500 eV cutoff energy and proportionally reduced 2 × 2 × 1 ***k***-point meshes. For these calculations, the PAW projection was performed in reciprocal space, and an auxiliary charge–density grid with 8× the density of points was used when evaluating the forces.

## Results and Discussion

### Structure and Energetics

**Figure 1:**
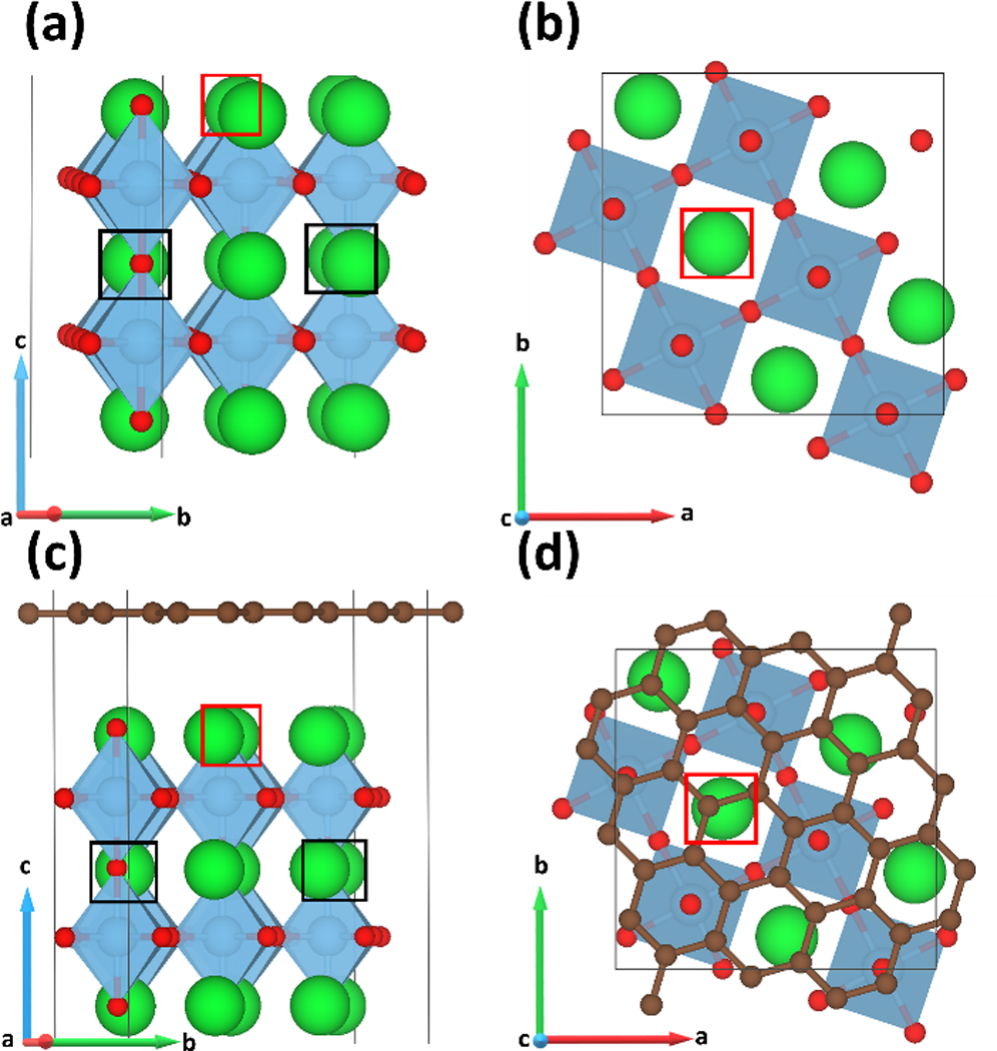
Surface structures investigated in this work. (a, b) SrTiO_3_ surface—(a) side and (b) top view. (c, d) graphene-decorated SrTiO_3_ surface—(c) side and (d) top view. The black and red squares mark the positions of La dopant atoms and charge-balancing Sr vacancies in the corresponding La-doped SrTiO_3_ surface models.

The optimized surface models investigated in this work are shown in Figure 1. Low-energy ion-scattering (LEIS) experiments have shown that perovskite surfaces display a dominant AO termination at elevated temperature,^69−72^ based on which we chose the SrO-terminated {001} surface of SrTiO_3_ as our starting point. An appropriate slab model was constructed from bulk cubic SrTiO_3_, consisting of 20 SrTiO_3_ and 5 SrO formula units (Figure 1). The additional 5 SrO units are required to form a complete monolayer, and the top and bottom surfaces of the slab models are therefore identical. A single-layer graphene model comprising 30 C atoms was then constructed and combined with the SrTiO_3_ surface, with the lattice vectors of both components redefined using the METADISE code^73^ to reduce the residual strain on the graphene sheet to ∼0.90%.^52^

Models of La-doped SrTiO_3_ with and without adsorbed graphene were further generated by introducing two Sr vacancies on each side of the corresponding SrTiO_3_ slabs and substituting four subsurface Sr atoms with La to maintain overall charge neutrality, which we found to be the most stable arrangement.^52^

The surface energies of the SrTiO_3_ and La-doped SrTiO_3_ slab models were calculated according to
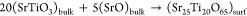
1
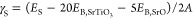
2
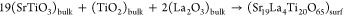
3
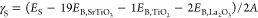
4where the *E*_S_ and *E*_B_ are the energies of the surface and bulk models, respectively, and *A* is the surface area. We calculate a γ_S_ of 1.76 J/m^2^ for SrTiO_3_ and 1.86 J/m^2^ for La-doped SrTiO_3_.

The graphene layer adsorbs to the SrTiO_3_ surfaces at a distance of 3.23 Å, in good agreement with the 3.19 Å distance obtained in the calculations by Zou *et al*.^74^ The graphene adsorption and interfacial adsorption energies were calculated as
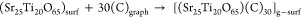
5
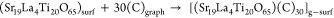
6
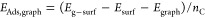
7
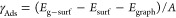
8where *E*_g-surf_ and *E*_surf_ are the energies of the graphene-coated and pristine surfaces, respectively, and *E*_graph_ is the energy of the graphene sheet with *n*_C_ = 30 carbon atoms. We note that our models of pristine graphene and the graphene-decorated surfaces have the same number of C atoms. We obtain *E*_Ads, graph_ = −0.01 and −0.05 eV per C atom for the SrTiO_3_ and La-doped SrTiO_3_ surfaces, respectively, which give the magnitude of the attachment energy for graphene to the oxide surfaces and indicate a small but energetically favorable adsorption. The interfacial adsorption energies were calculated to be −0.08 and −0.32 J/m^2^, respectively, for graphene adsorbed to pristine SrTiO_3_ and La-doped SrTiO_3_ surfaces. Such small energies are similar to the average binding energy of graphene onto the SrTiO_3_ (100) surfaces of 0.056 eV per C atom calculated by Zou *et al*.^74^ The difference in energy may be due to the lower level of strain in the graphene sheets in this work (0.9 vs 2.5% in the study in ref (74)).

Finally, the energies for La doping were calculated as
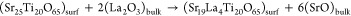
9
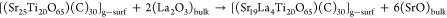
10
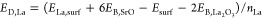
11where the *E*_B_ are the bulk energies of SrO and La_2_O_3_, *E*_La,surf_ and *E*_surf_ are the energies of the La-doped and pristine surfaces, respectively, and *n*_La_ is the number of La dopant atoms. We calculate an *E*_D,La_ of 0.57 eV per La atom for the SrTiO_3_ surfaces and 0.28 eV per La for the graphene-coated SrTiO_3_ (g-SrTiO_3_) surfaces, indicating that the energetic penalty for La doping is substantially reduced in the presence of graphene.

### Electronic Structure

We calculate direct and indirect band gaps of 2.0 and 1.65 eV for bulk SrTiO_3_ (Figure S2). The former may be compared to values of 2.74 eV using the LDA^75^ and 2.34 eV using the PBE GGA.^76^ Similarly, our calculated indirect gap can be compared to values of 2.14^75,77^ and 1.85 eV using the LDA^78^ and 1.99 eV using PBE.^76^ Our calculations deviate from the experimental values of 3.75 and 3.25 eV (direct/indirect) due to the well-known band gap underestimation inherent in (semi-)local DFT.^79^ The underestimation of our calculated band gaps compared to other computational literature is likely due to the inclusion of a van der Waals correction (DFT-D3); indeed, Holmström *et al*.^80,81^ calculated a band gap of 1.76–2.00 eV with PBE-D3, which is in keeping with our results. Despite the band gap underestimation, our calculations still correctly predict SrTiO_3_ to be a semiconductor and thus do not affect the qualitative analysis of the electronic structure, and, most importantly, the quantitative comparison between the slab models.

**Figure 2:**
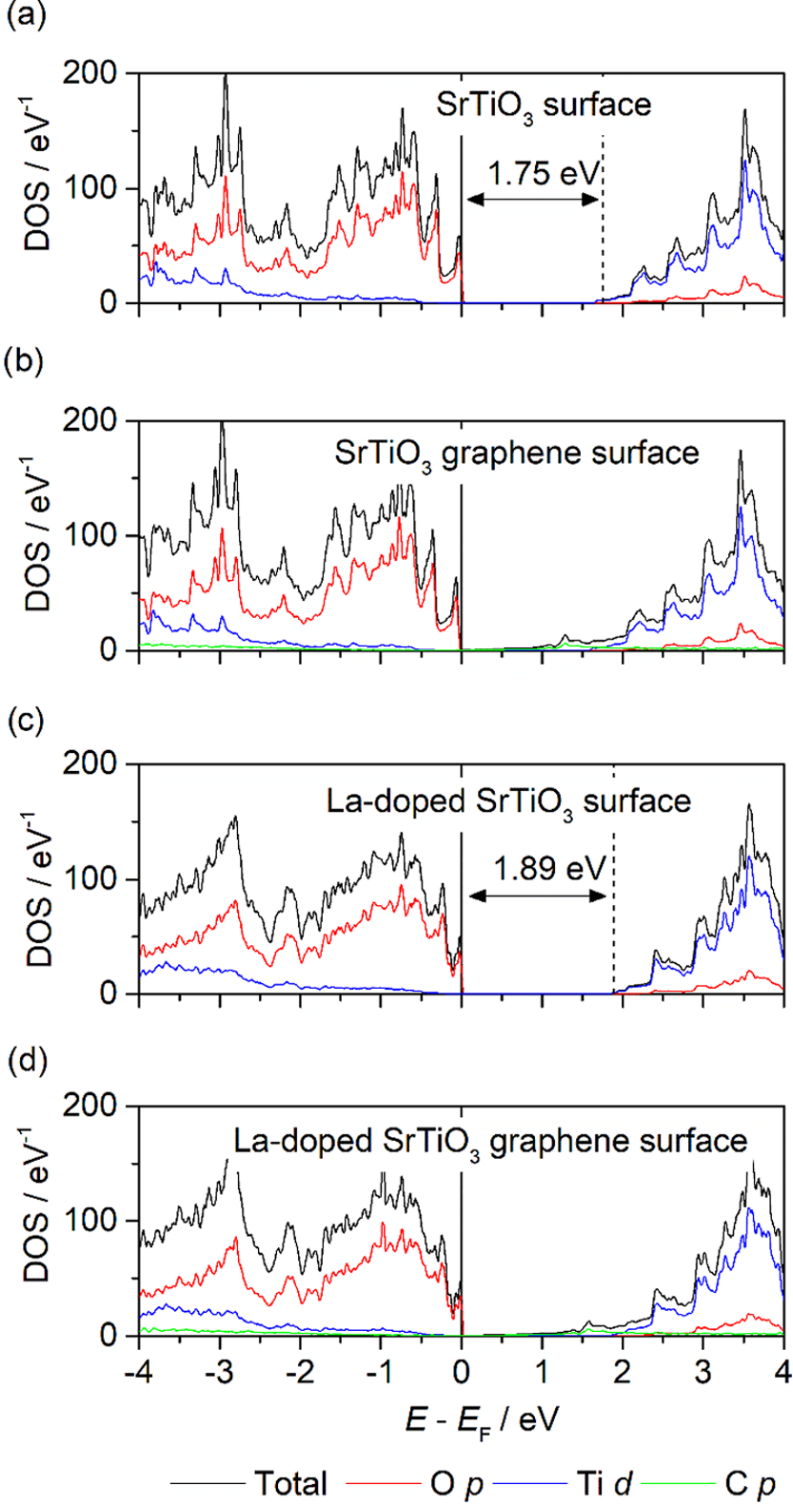
Total and orbital-projected electronic densities of states (DOS) from DFT calculations showing the composition of the valence and conduction bands in SrTiO_3_ and La-doped SrTiO_3_ surfaces with and without adsorbed graphene sheets.

The electronic density of states (DOS) of SrTiO_3_ and La-doped SrTiO_3_ surface models indicate them to be semiconductors. As for bulk SrTiO_3_, the surfaces are predicted to have an indirect band gap (Figures S3 and S4). The orbital-projected partial density of states (Figure 2a,c) shows an asymmetric DOS with sharp peaks in the valence band. At the PBE-D3 level of theory, the calculated band gaps for SrTiO_3_ and La-doped SrTiO_3_ are 1.75 and 1.89 eV, respectively. These are in line with previous calculations on the SrO-terminated (001) surface, which estimated band gaps of 1.72–2.01 eV^80^ (PBE-D3) and 1.86 eV (LDA).^78^ In agreement with both theory and experiment, we find that the valence band maximum and conduction band minimum are dominated by O 2p and Ti 3d states, respectively.^79^ The addition of graphene to the surface introduces C 2p states into the SrTiO_3_ band gap (Figure 2b,d). However, the contribution of these states at the Fermi level is relatively small, and as such that the composite could be classified as a semimetal, that is, graphene does not induce metallic behavior in bulk SrTiO_3_, but rather introduces gap states that allow for metallic conduction with a small number of carriers. Previous studies using the (bare) PBE functional predicted a similar introduction of C 2p states at the Fermi level when SrTiO_3_ is composited with porous graphene (i.e., with carbon vacancies), irrespective of the concentration of pores.^50^

### Electronic Transport

It is clear from Figure 2 that the interaction of graphene with SrTiO_3_ surfaces intrinsically alters the electronic structure and as such is expected to impact upon the electronic transport and thermoelectric properties. The three crucial properties controlled by the electronic structure are the Seebeck coefficient *S*, the electrical conductivity σ, and the electronic part of the thermal conductivity κ_el_.

**Figure 3:**
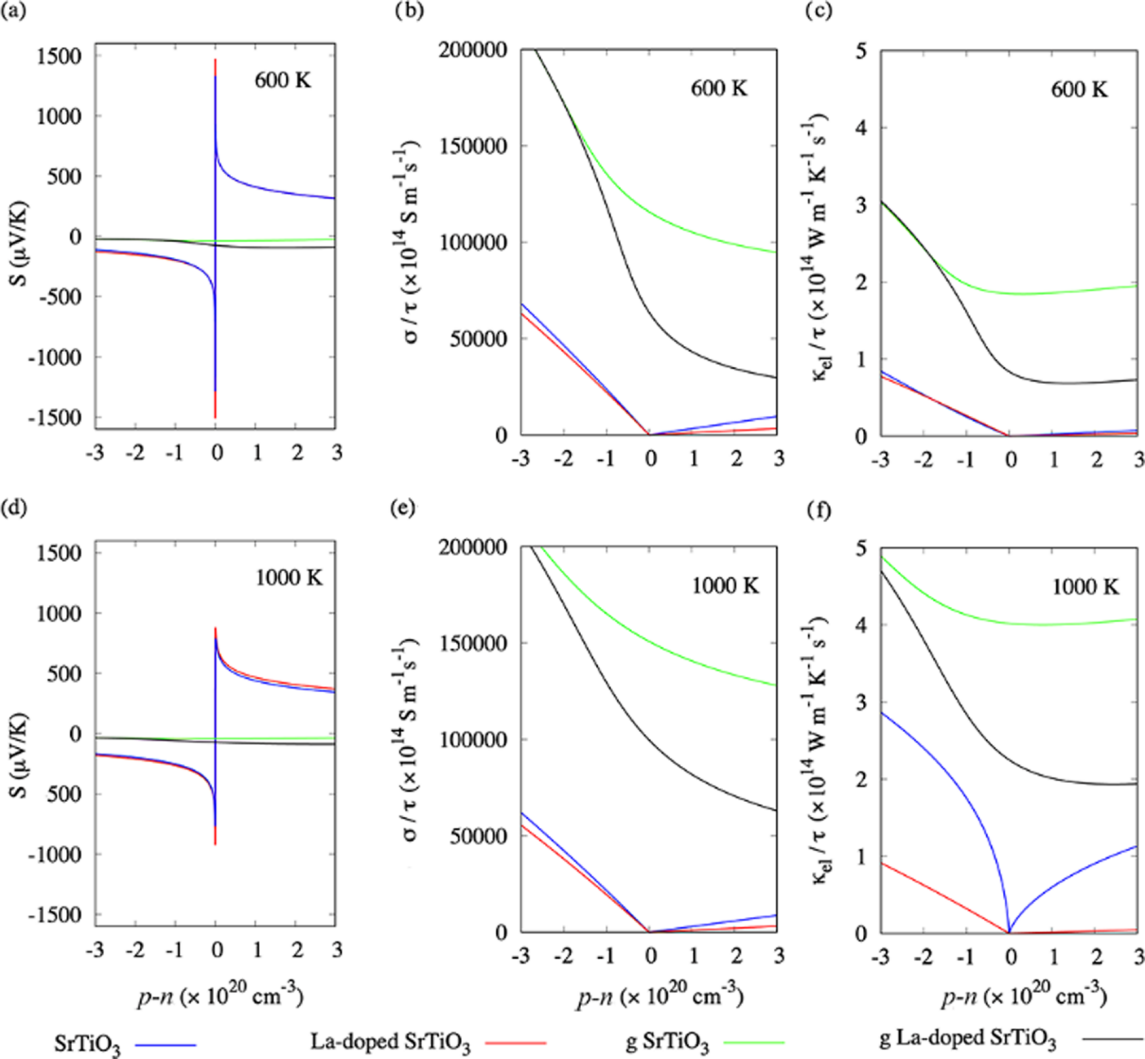
Seebeck coefficient *S* (a, d), electrical conductivity σ/τ (b, e), and electronic thermal conductivity κ_el_/**τ** (c, f) as a function of carrier concentration p–n for pristine and graphene-adsorbed SrTiO_3_ and La-doped SrTiO_3_ surfaces at *T* = 600 (a–c) and 1000 K (d–f), calculated using the BoltzTraP code.

Figure 3 shows calculations of these three properties as a function of carrier concentration for the pristine and graphene-adsorbed surfaces. The same properties as a function of chemical potential are presented in Figure S10. Figure 3a compares the Seebeck coefficients of the pristine SrTiO_3_ and La-doped SrTiO_3_ surfaces as a function of carrier concentration. Within the constant relaxation-time approximation (CRTA) used by BoltzTraP, σ and κ_el_ are determined with respect to an unknown electron relaxation time τ, while *S* is independent of this parameter. Within this model, we obtain Seebeck coefficients of 1 333 and 1 475 μV/K at 600 K for the SrTiO_3_ and La-doped SrTiO_3_ surfaces, respectively (Figure 3a). Comparable values of 1342 and 1498 μV/K are obtained using BoltzWann (Figure S7). A general feature of the DOS of both models is an asymmetry arising from sharper features at valence band edges than at conduction band edges. For an intrinsic (undoped) semiconductor, where no charge carriers are present (p–n = 0), the Seebeck coefficient is governed solely by the gradient of the DOS at the band edges. In undoped SrTiO_3_, the larger gradient of the DOS at the valence band edge compared to the conduction band edge leads to a larger p-type contribution and a positive *S* (Figure S9). At higher temperatures, the smoothing out of the Fermi–Dirac distribution reduces the asymmetry in the DOS and *S* becomes negative, indicating n-type conductivity.^82^ The larger Seebeck coefficient of the La-doped SrTiO_3_ surface compared to the SrTiO_3_ surface is consistent with the steeper states in the DOS of the former (Figure 2).

**Figure 4:**
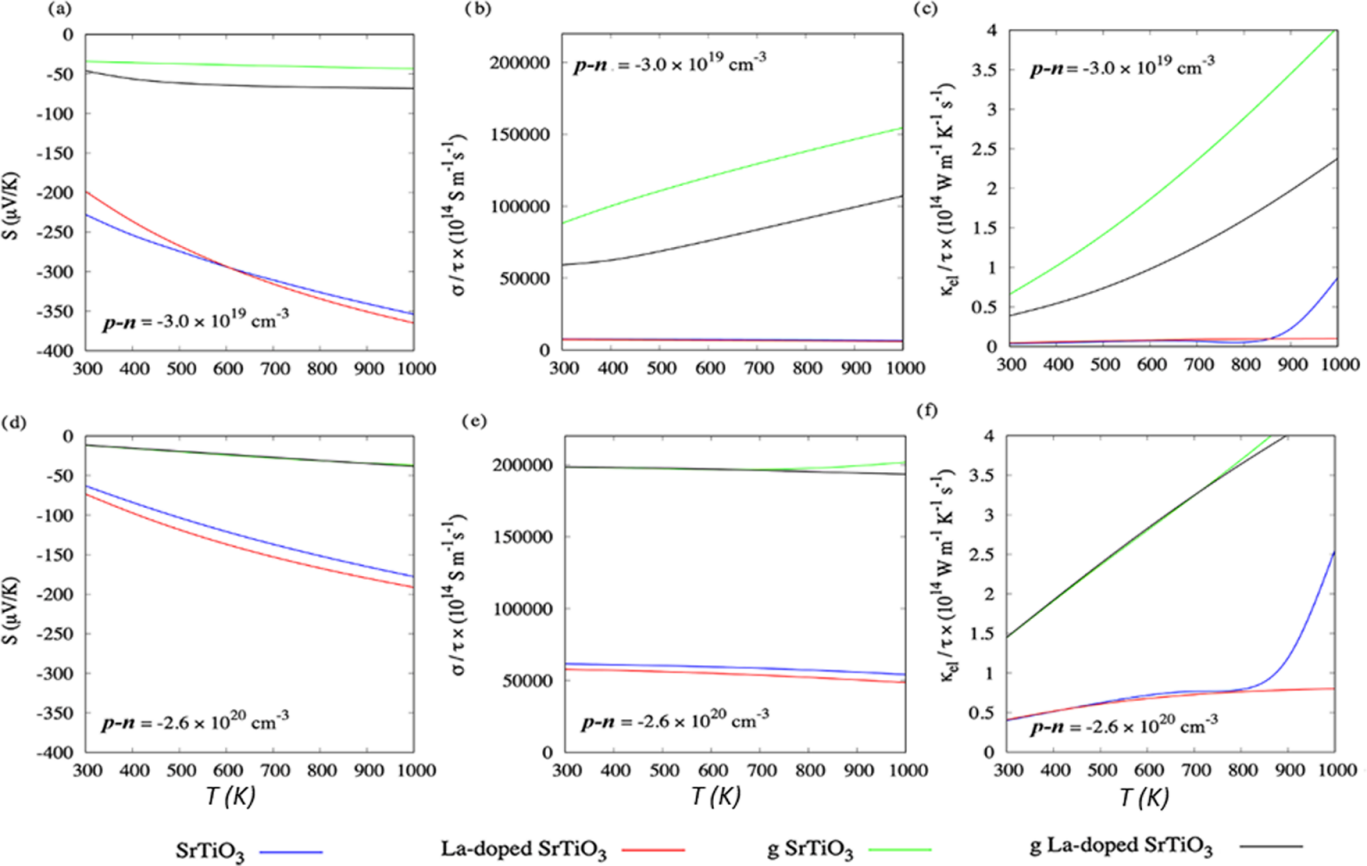
Seebeck coefficient *S* (a,d), electrical conductivity σ/τ (b,e), and electronic thermal conductivity κ_**el**_/τ (c,f) as a function of temperature for pristine and graphene-adsorbed SrTiO_3_ and La-doped SrTiO_3_ surfaces, with carrier concentrations of p–n = −3.0 × 10^19^ cm^–3^ (a–c) and −2.6 × 10^20^ cm^–3^ (d–f), calculated using the BoltzTraP code.

These values represent the maximum theoretical value that could be obtained for ideal, defect-free compositions. In reality, all materials contain defects that promote n or p-type conductivity, and the carrier concentrations are therefore heavily dependent on the material preparation. Both bulk and thin-film SrTiO_3_ show n-type conductivity,^83,84^ so we calculated the carrier concentrations relative to the bottom of the conduction band. This method is valid within the rigid-band approximation^65^ and has been used on similar perovskite systems. We obtain a carrier concentration of ∼10^20^ cm^–3^ for bulk SrTiO_3_ (Figure S6) and about 2.6 × 10^20^ cm^–3^ for the SrTiO_3_ surface model, which is in line with the ∼3.5 × 10^20^ cm^–3^ reported by Ravichandran *et al*. for undoped SrTiO_3_ thin films.^84^ Based on recent experiments,^13^ we also report values of *S* at carrier concentrations of 3 × 10^19^ cm^–3^. Figure 4a shows the Seebeck coefficients obtained for the surface models using BoltzTraP, and comparisons with BoltzWann are given in Figures S5–S7. The absolute value of the Seebeck coefficient increases with temperature, as is observed in bulk SrTiO_3_ at a fixed carrier concentration, and has previously been reported for bulk^85^ and thin-film samples.^84^

The electronic structures in Figure 2 show that the addition of graphene introduces C 2p states into the SrTiO_3_ band gap, resulting in a semimetallic electronic structure for both graphene-adsorbed surfaces. As shown in Figures S10a, 3a, and 4a, this results in an extremely low Seebeck coefficient at 600 K, with an order of magnitude difference in the maximum absolute values between the pristine and graphene-adsorbed surfaces. While inconsistent with the findings of Lin *et al*.,^13^ this is most likely due to the effective concentration of graphene in our models—whereas we have an effectively infinite graphene layer adsorbed onto the surfaces of the perovskite material, which would correspond to an ∼8 wt % composite, the highest concentration used in the experimental work is a much smaller 1 wt %. However, we can infer from the discrepancy that the electrical performance of these composite TEs is likely to be highly sensitive to the amount of graphene present and also its quality in terms of flake size and defect concentration, as these would strongly influence both the electrical properties of the graphene sheets and their interaction with the perovskite surfaces. Introducing high concentrations of graphene in graphene-oxide composites would lead to the thermoelectric properties being driven mostly by the graphene component, and from this perspective, our results are qualitatively in line with the Seebeck coefficients predicted for graphene from tight-binding models.^86^

At higher carrier concentrations, *S* decreases with temperature (Figure 4a,d), implying that the oxide component retains some influence on the properties of the composite. This is opposite to what is seen for carbon nanoribbons interacting with SrTiO_3_ surfaces, where *S* instead increases with temperature in the composite material.^52^

As noted above, solving the Boltzmann transport equations within the constant relaxation-time approximation yields the electrical conductivity σ and electronic thermal conductivity κ_el_ with respect to an unknown electron scattering time τ. τ incorporates complex physical phenomena including electron–phonon coupling and cannot, at present, practically be calculated from the first principles. For completeness, we plot these two properties as a function of carrier concentration (Figure 3), chemical potential (Figure S10), and temperature (Figure 4) and again observe substantially different behavior for the pristine and graphene-adsorbed systems. As noted above, the graphene-adsorbed systems have semimetallic character, since electronic states cross the Fermi level in graphene-adsorbed models, and we predict p-type conduction. The change from semiconducting to metallic behavior has a large effect on the transport properties, resulting in an increased σ and κ_el_ together with a reduced *S*, since the latter is inversely related to the number of free carriers.^87^

### Lattice Dynamics and Thermal Transport

The lattice component of the thermal conductivity κ_latt_ can be calculated using the second-order (harmonic) and third-order (anharmonic) force constants. However, obtaining the latter is computationally very expensive and is prohibitive for large systems such as our surface models. For this reason, we chose instead to examine the atom-projected phonon density of states (PDOS), the weighted two-phonon joint density of states (wJDOS), and the mode group velocities, all of which can be computed using the second-order force constants, to assess how changes in the phonon spectrum due to the doping and graphene adsorption are likely to impact the heat transport.^13^

**Figure 5:**
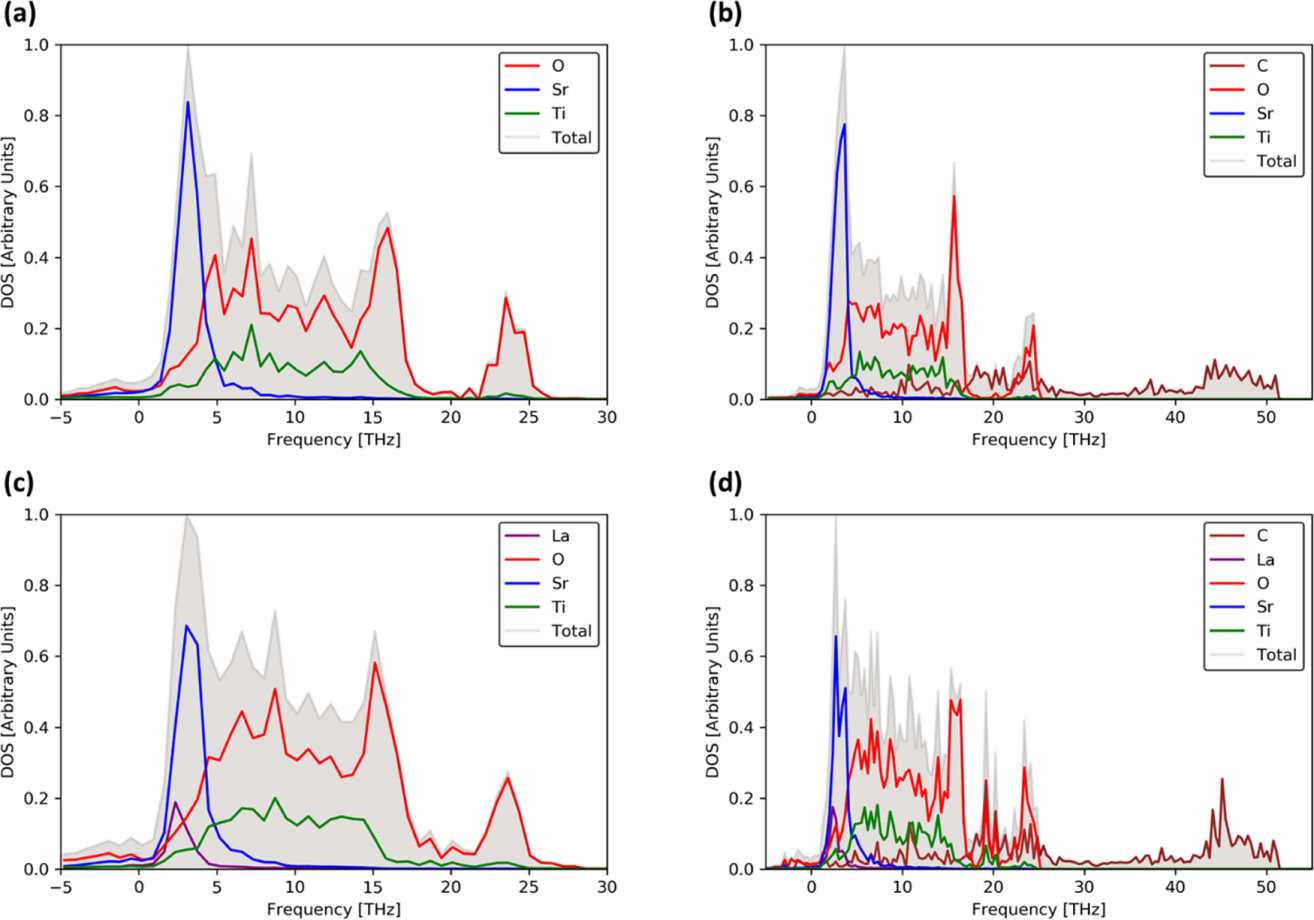
Atom-projected partial phonon density of states (PDOS) of the SrTiO_3_ surface model (a), the SrTiO_3_ surface with adsorbed graphene (b), the La-doped SrTiO_3_ surface model (c), and the La-doped SrTiO_3_ surface with adsorbed graphene (d).

Figure 5 compares the atom-projected phonon DOS (PDOS) curves of the four surface models. The PDOS curves of La-doped surfaces show large contributions from La at low frequencies, suggesting that localized “rattling” modes involving La^3+^ cations could in principle couple strongly to low-frequency acoustic modes to suppress transport through the TiO_6_ octahedral framework. There is also a significant contribution from C to the low-frequency DOS in the graphene models, which likewise suggests that the graphene and SrTiO_3_ modes may also couple to suppress the thermal transport of both components.

Within the single-mode relaxation-time approximation (RTA) to the phonon Boltzmann transport equation, the lattice thermal conductivity tensors **κ**_latt_(*T*) can be written as a sum of contributions **κ**_λ_(*T*) from the individual phonon modes λ as
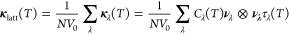
12where *C*_λ_ are the modal heat capacities, **ν**_λ_ are the group velocities, *V*_0_ is the volume of the unit cell, *N* is the number of reciprocal-space wavevectors (**q**) included in the sum over the Brillouin zone, and τ_λ_ are the phonon lifetimes given by
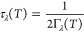
13where Γ_λ_ are the phonon linewidths obtained as the self-energy from third-order perturbation theory. *C*_λ_ and **ν**_λ_ are calculated within the harmonic approximation as
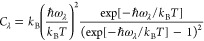
14
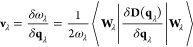
15where ω_λ_ are the phonon frequencies, **W**_λ_ are the corresponding mode eigenvectors, and **D**(**q**) is the dynamical matrix for the wavevector **q**.

**Figure 6:**
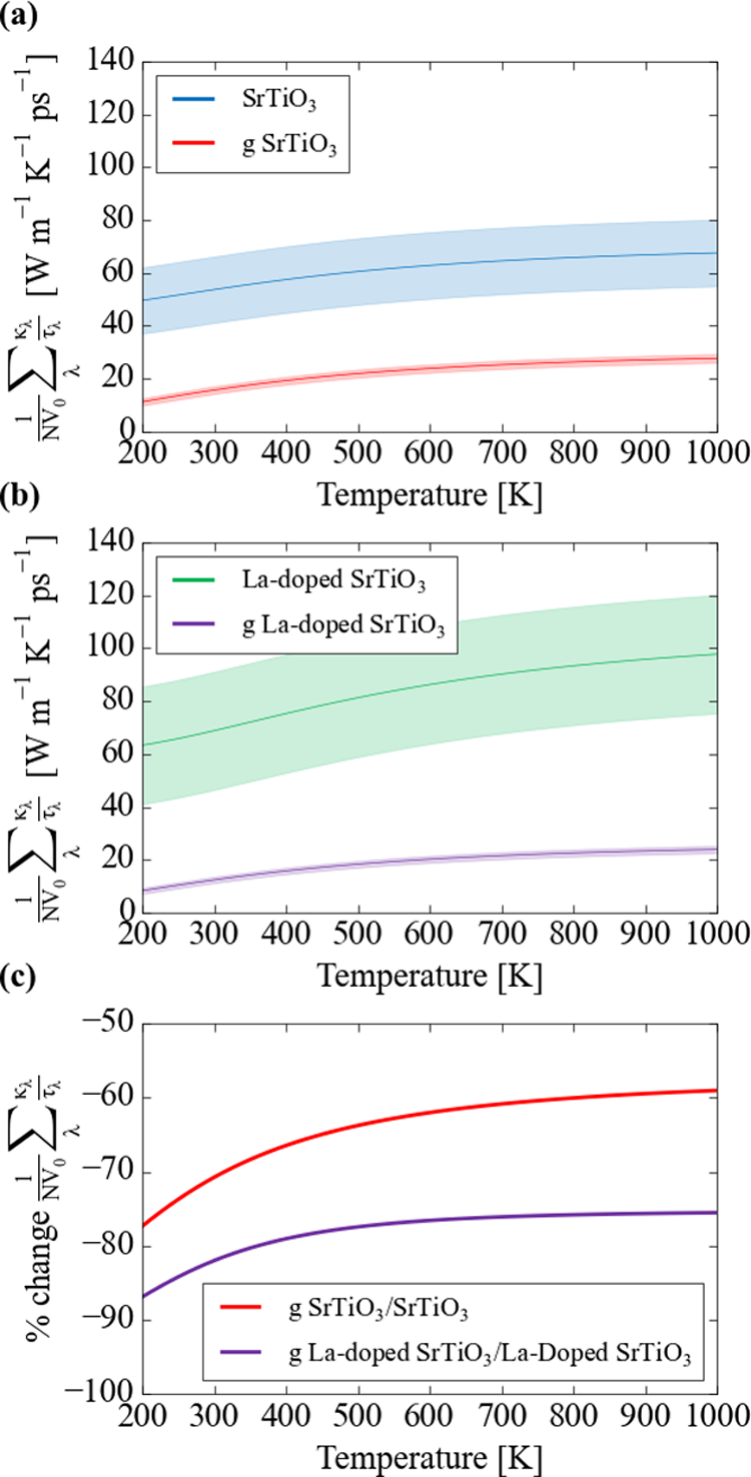
Calculated **κ**_latt_/τ^CRTA^ for in-plane transport in (a) pristine and graphene-adsorbed SrTiO_3_ and (b) La-doped SrTiO_3_ and graphene-adsorbed La-doped SrTiO_3_ surfaces. The shaded areas represent the calculated error due to erratic convergence of the function in eq 16 with respect to the Brillouin-zone sampling mesh (Figure S12). Plot (c) shows the calculated percentage change in the average **κ**_latt_/τ^CRTA^ due to the addition of graphene at the two surfaces.

Although the calculation of τ_λ_ (*T*) in eq 13 is computationally prohibitive, we can follow the approach of Tang and Skelton^88^ and compute the sum over harmonic terms to obtain **κ**_latt_ with respect to an unknown relaxation time τ^CRTA^ as
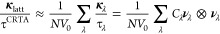
16where the modal terms are the same as defined in eq 12. In the following, we consider the diagonal components of κ_latt_/τ^CRTA^ for in-plane transport 1/2 (*xx* + *yy*), after rotating the tensors to align with the equivalent directions in bulk STO (see Supporting Information). This analysis shows that one would expect a reduction in **κ**_latt_ on the order of 60–90% by adsorbing graphene at the surface, based on the changes in the model heat capacities and group velocities (Figure 6). We note that we had difficulty converging the function in eq 16 with respect to the **q**-point sampling mesh (see the Supporting Information), although despite this, we still predict a reduction in the **κ**_latt_ due to the graphene adsorption. This reduction is more pronounced in the case of lanthanum doping, with a predicted average reduction of 80% over the temperature range 400–1000 K compared to a 60–70% reduction without doping. This suggests a synergy between La doping and graphene addition in suppressing the thermal transport. A possible mechanism for this is that graphene increases the “effective mass” of the bulk STO modes and therefore reduces the frequency dispersion and “depresses” the mode group velocities. This would be consistent with the stronger graphene adsorption energies calculated for the La-doped surface. The predicted reduction in thermal conductivity supports the experimental work by Lin *et al.*,^13^ which demonstrated a widening of the operating temperature window for graphene/La-doped SrTiO_3_ samples to well below the standard operating temperature of the undoped material.

We also note in passing that the average **κ**_latt_/τ^CRTA^ functions suggest that La doping would lead to changes in the harmonic terms in eq 15 that would increase **κ**_latt_. However, the poor convergence behavior means this finding may not be reliable. Moreover, as suggested by the phonon DOS, it is possible that “rattling” of the La ions would counteract the increase in the harmonic terms by reducing the phonon lifetimes, leading to an overall decrease in the thermal conductivity. We explore this point further in the following.

The Γ_λ_ in eq 13 are computed as a sum of contributions from energy- and momentum-conserving three-phonon scattering processes between phonon triplets consisting of a reference mode λ and two interacting modes λ′ and λ″. Computing the three-phonon interaction strengths Φ_λλ′ λ″_, which capture the strength of the physical coupling between the modes, requires the third-order force constants, and obtaining these represents a substantial computational workload. If the coupling strength can be averaged over interacting modes λ′ and λ″, however, Γ_λ_ can be approximated as^68^
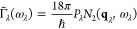
17

**Figure 7:**
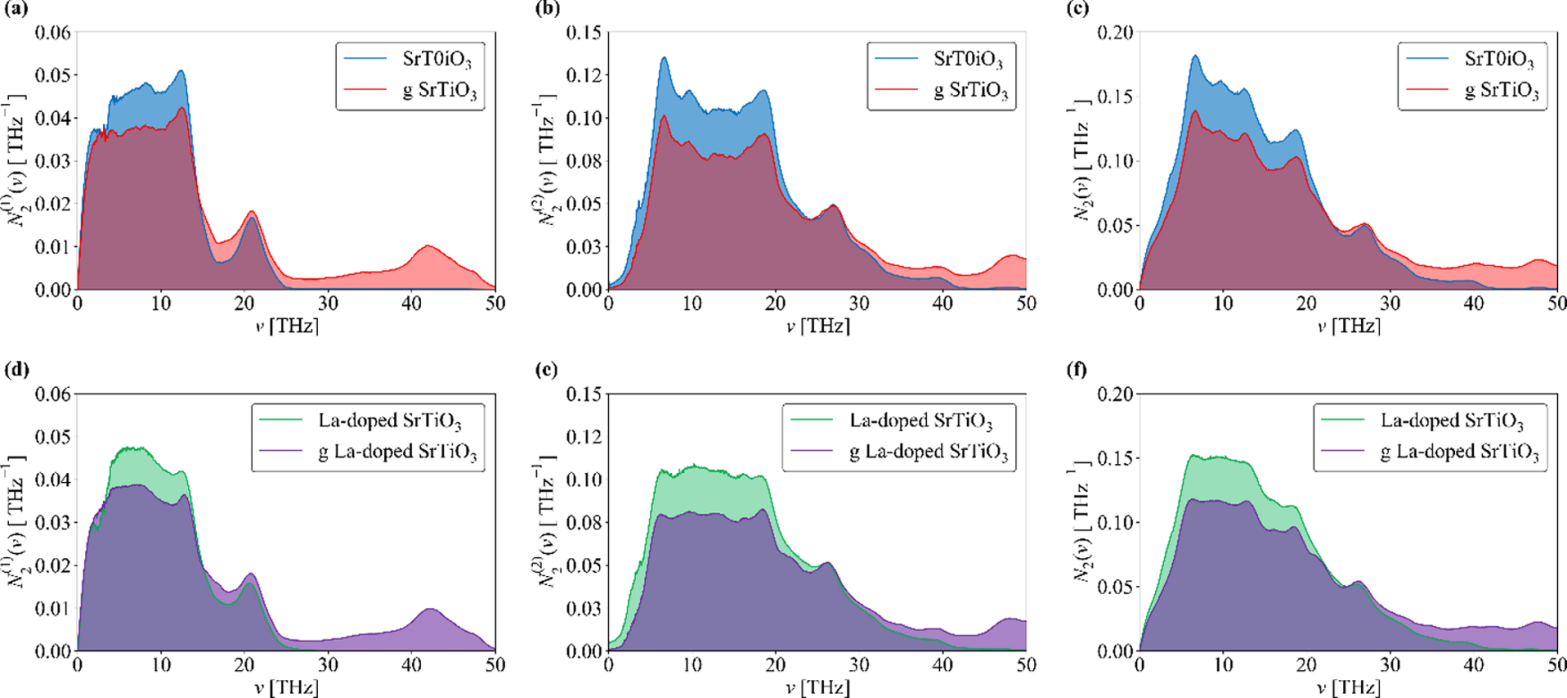
Weighted joint two-phonon density of states (wJDOS) for (a, d) Class 1 events (*N̅*_**2**_^(**1**)^), (b, e) Class 2 events (*N̅*_**2**_^(**2**)^), and (c, f) both types of event (*N̅*_2_) for pristine and graphene-coated SrTiO_3_ surfaces with and without La doping.

*P*_λ_ is the averaged phonon–phonon interaction strength defined as
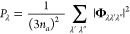
18where *n*_a_ is the number of atoms in the primitive cell, and hence 3*n*_a_ is the number of phonon bands at each wavevector **q**. *N*_2_ (**q**, ω) is the weighted two-phonon density of states (wJDOS) and is a sum of two functions for collision (Class 1) and decay processes (Class 2)
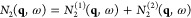
19
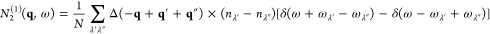
20
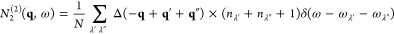
21Here, the two functions Δ and δ enforce the conservation of (crystal) momentum and energy, respectively, and *N*_2_^(1)^ and *N*_2_^(2)^ count the number of energy- and momentum-conserving scattering processes for a reference mode with wavevector **q** and frequency ω. For making comparisons between systems, it is useful to further average the wJDOS functions over wavevectors to obtain functions of frequency only, i.e.,
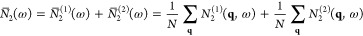
22The phonon line widths (inverse lifetimes) can thus be separated into contributions from how strongly the modes interact, and contributions from the conservation of energy, due to the shape of the phonon spectrum, which are captured by the wJDOS.^68,89^ Unlike *P*_λ_ in eq 18, *N̅*_2_(ω) can be computed from the harmonic phonon frequencies. Figure 7 shows the calculated *N̅*_**2**_^(**1**)^ and *N̅*_**2**_^(**2**)^ for the SrTiO_3_ surface with and without La doping and adsorbed graphene, generated using a sampling mesh with 8 × 8 × 1 wavevectors (convergence tests are shown in Figure S13). To allow for comparison between systems, we have normalized the wJDOS functions by (3*n*_a_)^2^, which is usually folded into the *P*_λ_ term in the expression for the approximate linewidth (cf. eq 18).

This method allows us to assess whether changes to the harmonic phonon spectrum are likely to increase/decrease the phonon linewidths and thereby decrease/increase the lifetimes. Since the thermal conductivity is proportional to the lifetimes, this can be a useful means to qualitatively compare how different dopants might affect the heat transport without the computational expense of a full third-order calculation. Peaks in *N̅*_2_(ω) at low frequencies are potentially indicative of enhanced acoustic-mode broadening; since these modes typically make the largest contributions to the thermal conductivity, this would suppress the overall heat transport.^89^ However, Figure 7 shows that adsorption of graphene tends to decrease the *N̅*_2_(ω) at low frequencies. Similarly, comparing between the pure SrTiO_3_ and La-doped systems suggests that the doping also has little effect on the wJDOS. This suggests that either the changes to the group velocities noted above are the dominant mechanism for the reduced thermal conductivity of the composites observed by Lin *et al*.^13^ or that changes to the frequency spectrum/wJDOS are secondary to changes in interaction strengths in reducing the phonon lifetimes in the composites and doped samples. In particular, our calculations do not indicate significant changes in the harmonic terms between the pristine and La-doped surface models without graphene, and the wJDOS functionals also seem to be generally smaller, so we might infer from this that any reduction in the thermal conductivity on doping may be attributed to stronger phonon–phonon interactions. To investigate this further would require explicit computation of the third-order force constants, which, as noted above, is presently not practical.

## Conclusions

We have performed a detailed study of how the adsorption of graphene at the surface of SrTiO_3_ and La-doped SrTiO_3_ affects the electronic structure, transport properties, structural dynamics, and lattice thermal conductivity. Based on our results, we draw three main conclusions from our study.

First, there is a thermodynamic driving force for graphene to adsorb to the surfaces of SrTiO_3_ and La-doped SrTiO_3_, yielding favorable adsorption energies.

Second, we find that the adsorption of graphene introduces C 2p states into the SrTiO_3_ band gap, which would account for the reduced Seebeck coefficient observed by Lin *et al*.,^13^ but which is likely mitigated experimentally by using smaller amounts of graphene in the composites. This highlights the sensitivity of the electrical properties to the amount and quality of the graphene in the composite and indicates that controlling this may be a simple route to fine-tuning the thermoelectric properties. Our calculations predict that extended graphene layers will induce a change from semiconducting to semimetallic behavior. However, understanding whether this behavior is localized at interfaces or extends into the bulk will require input from both experiments and modeling to elucidate the impact of different graphene concentrations on the properties of the composite.

Finally, analysis of the phonon spectra shows a remarkable reduction in the phonon group velocities on graphene adsorption, which we predict to lead to decreases of 60–90% of the bulk lattice thermal conductivity. This effect appears to be enhanced by La doping. Furthermore, analysis of the phonon spectra suggests that both La doping and graphene adsorption introduce low-frequency modes into the density of states. The former, in particular, hints at low-frequency “rattling” modes in La-doped systems that could couple to acoustic modes and suppress the phonon lifetimes. While calculations of the two-phonon density of states do not suggest enhanced acoustic-mode broadening in La-doped or graphene-adsorbed systems, it is possible that a reduction in the mode lifetimes may instead be facilitated by stronger phonon–phonon interactions. This can potentially be investigated in the future by explicit calculation of the third-order force constants.

## Supporting Information Available

A detailed description of electronic transport calculations performed using the BoltzWann code and comparison to results obtained with BoltzTraP; additional data from BoltzTraP transport calculations; conversion of the in-plane thermal conductivity of the rotated slab model to the bulk SrTiO_3_ axes; and convergence of the harmonic CRTA function in eq 16 and wJDOS functions in eq 22 with respect to the **q**-point sampling mesh (PDF)

## Notes

Raw data from this study is available from the authors on reasonable request.
