# Supplementary material for: Unraveling the Impact of Graphene Addition to Thermoelectric SrTiO3 and La-Doped SrTiO3 Materials: A Density Functional Theory Study
Source: ACS Appl Mater Interfaces. 2021 Aug 18;13(34):41303–14. doi: 10.1021/acsami.1c10865 (PMC8414480; doi:10.1021/acsami.1c10865)
Supplement: Supplementary file 1 — am1c10865_si_001.pdf [file am1c10865_si_001.pdf]

Unravelling the Impact of Graphene Addition to Thermoelectric $SrTiO_3$ and La-doped $SrTiO_3$ Materials: A Density Functional Theory Study

Supporting Information

Joshua Tse,[1,§] Alex Aziz,[2,§] Joseph M. Flitcroft,[1,3,§] Jonathan M. Skelton,[3,*] Lisa J. Gillie,[1] Stephen C. Parker,[4] David J Cooke[1] and Marco Molinari[1,*]

1. Department of Chemical Sciences, University of Huddersfield, Queensgate, Huddersfield, HD1 3DH, UK.

2. Department of Chemistry, Queen Mary University of London, Mile End Road, London, E1 4NS, UK.

3. Department of Chemistry, University of Manchester, Oxford Road, Manchester M13 9PL, UK.

4. Department of Chemistry, University of Bath, Bath BA2 7AY, UK.

* E-Mail: m.molinari@hud.ac.uk and jonathan.skelton@manchester.ac.uk

[§] These authors contributed equally to this work.

Electronic Transport Calculations

We performed transport calculations on the pristine and La-doped $SrTiO_3$ surface-slab models using the BoltzWann code[1,2] with an interpolated 50 × 50 × 1 ***k***-point mesh. Due to the large number of bands and the semi-metallic nature of the surfaces with adsorbed graphene, we were not able to perform BoltzWann calculations on these models.

During the BoltzWann calculations, transport coefficients were calculated using maximally localized Wannier functions (MLWFs), which can be used for Wannier interpolation of the band energies onto a fine ***k***-point sampling mesh.[3] Test calculations on bulk $SrTiO_3$ showed that Wannier projections onto the oxygen *p* and titanium *d* orbitals using atom-centered Gaussian-type orbitals yielded the best MLWFs. In each case the final spreads for the WFs were < 1 Å with convergence to < 10^{-5} Å^2, and all the WFs were real. Figure S1 shows example Wannier functions obtained for the $SrTiO_3$ surface slab model.

The disentanglement method was used to freeze the bands around the Fermi level.[4] In this region of the band structure the Bloch states and therefore the bands are preserved, whereas in the outer energy window the Bloch states can change depending on the choice of unitary transformation. The disentanglement window was chosen to be between -6 eV below the Fermi level and ~1 eV above the lowest-energy conduction band. The bands contained in the outer window are not relevant for transport properties as they lie more than a few k_BT from the Fermi level.[2,5] For bulk $SrTiO_3$, projections onto the Ti *d* and O *p* orbitals give 14 WFs compared to the 15 bands calculated with DFT. Figures S2-S4 compare the band structures for bulk $SrTiO_3$ and the $SrTiO_3$ and La-doped $SrTiO_3$ slab models obtained by explicit computation of the band energies at strings of ***k***-points along the band path and by Wannier interpolation.

Figures S5 and S6 compare the calculated Seebeck coefficients of bulk $SrTiO_3$ obtained using both methods as a function of carrier concentration at T = 600 and 1000 K, and as a function of temperature at a fixed carrier concentration of 10^{20} cm^{-3}, corresponding to the chemical potential at the bottom of the conduction band. Similarly, Figures S7 and S8 compare the calculated Seebeck coefficients of the $SrTiO_3$ and La-doped $SrTiO_3$ surface models as a function of carrier concentration at 600 and 1000 K and as a function of temperature at a carrier concentration of -2.6×10^{20} cm^{-3}, corresponding to *n*-type doping.

Finally, Figures S9 and S10 show additional calculations performed using BoltzTrap to investigate the Seebeck coefficient of bulk $SrTiO_3$ at zero carrier concentration (Figure S9) and the Seebeck coefficient, electrical conductivity and electronic thermal conductivity as a function of carrier concentration at 300 K, and as a function of chemical potential at 300, 600 and 1000K for pristine and graphene-adsorbed $SrTiO_3$ and La-doped $SrTiO_3$ surfaces (Figure S10).

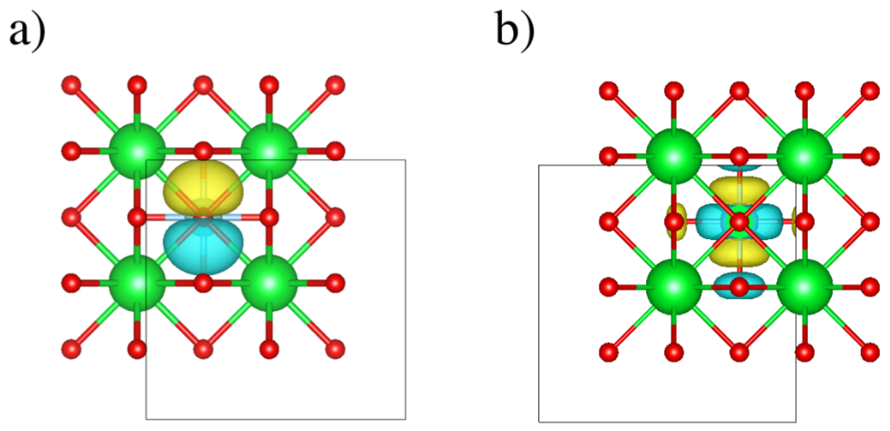

Figure S1. Real-space plots of representative MLWFs obtained for the $SrTiO_3$ surface slab model, generated using the VESTA software.[6] (a) *p*-type projection centered on an oxygen atom (red). (b) *d*-type projection centered on a titanium atom (bright green). Strontium atoms are shown in dark green.

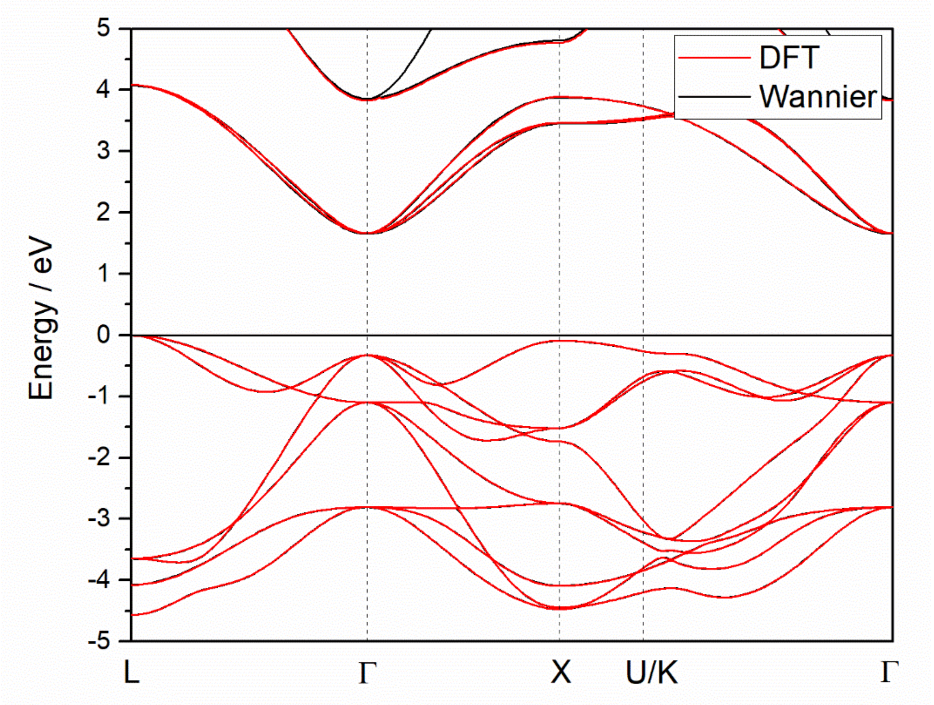

Figure S2. Comparison of the band structures of bulk $SrTiO_3$ obtained by explicit computation of the electronic band energies at strings of ***k***-points (red lines) and by Wannier interpolation with 14 Wannier functions (black). Both calculations predict the same indirect band gap of 1.65 eV.

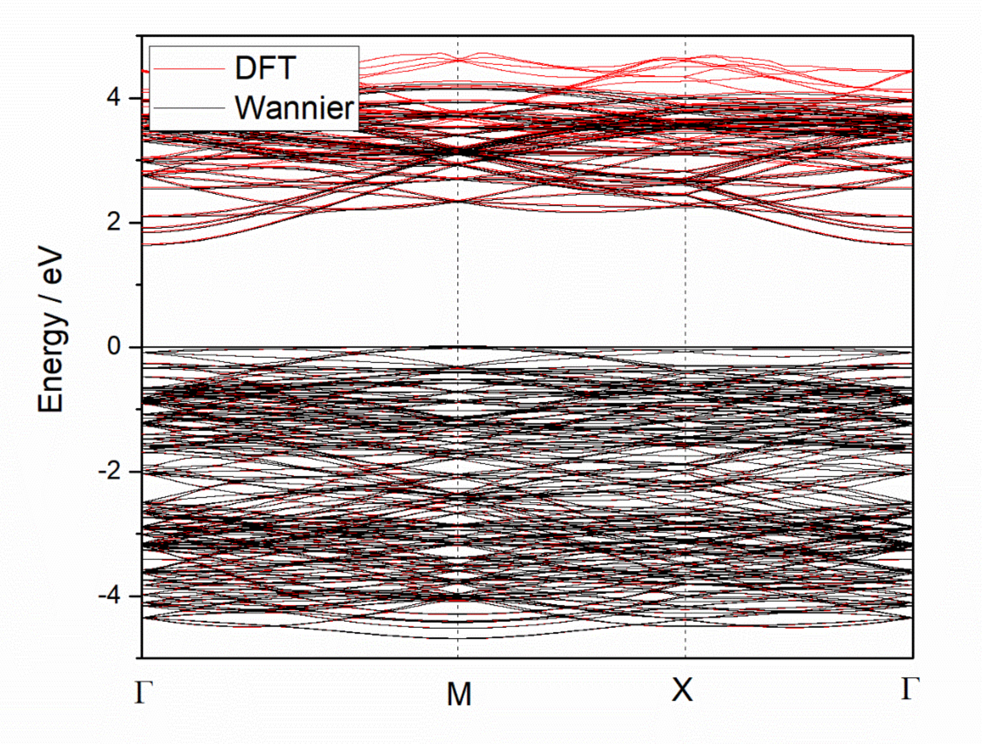

Figure S3. Comparison of the band structures of the $SrTiO_3$ surface slab model obtained by explicit computation of the electronic band energies at strings of ***k***-points (red lines) and by Wannier interpolation (black).

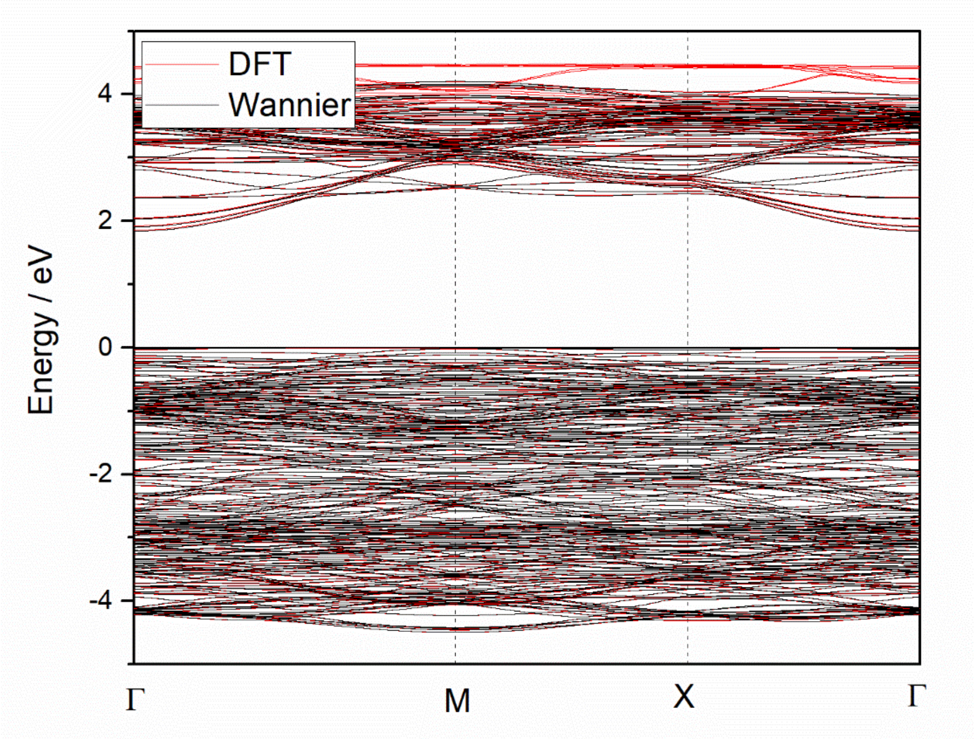

Figure S4. Comparison of the band structures of the La-doped $SrTiO_3$ surface slab model obtained by explicit computation of the electronic ban energies at strings of ***k***-points (red lines) and by Wannier interpolation (black).

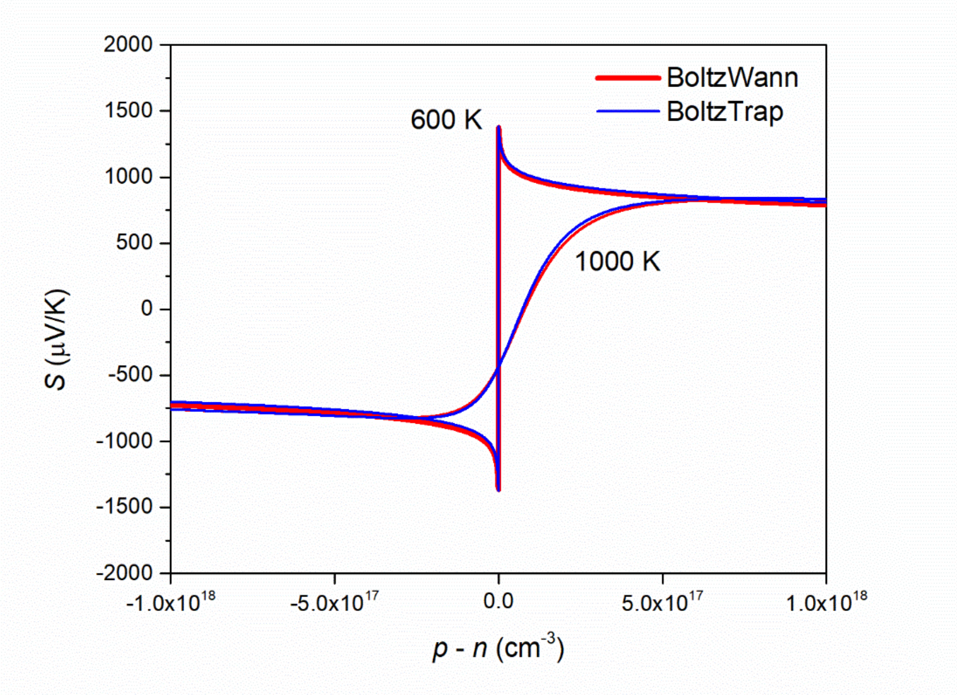

Figure S5. Comparison of the Seebeck coefficient of bulk $SrTiO_3$ as a function of carrier concentration obtained using BoltzTraP and BoltzWann at 600 and 1000 K. The plots show excellent agreement, indicating that our choice of Wannier functions is suitable. Experimentally, $SrTiO_3$ is intrinsically an *n*-type semiconductor with a negative Seebeck coefficient, corresponding to a negative *p*-*n*.[7]

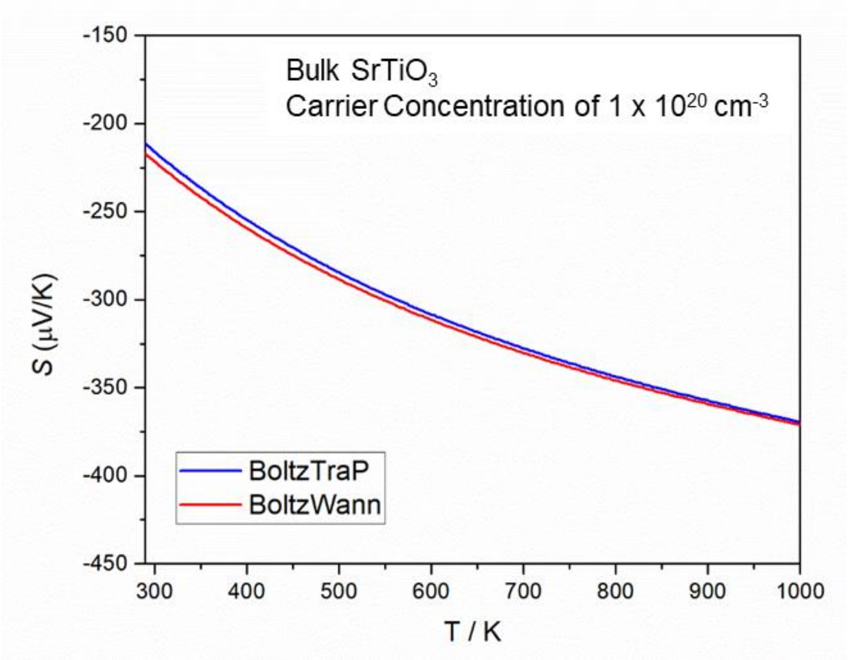

Figure S6. Comparison of the Seebeck coefficient of bulk $SrTiO_3$ as a function of temperature for a fixed carrier concentration of 10^{20} cm^{-3}, corresponding to the chemical potential at the bottom of the conduction band, obtained using the BoltzTraP and BoltzWann codes. Both the calculated Seebeck coefficient and its increase in magnitude as a function of temperature, in agreement with other theoretical modelling and experiments.[8–10]

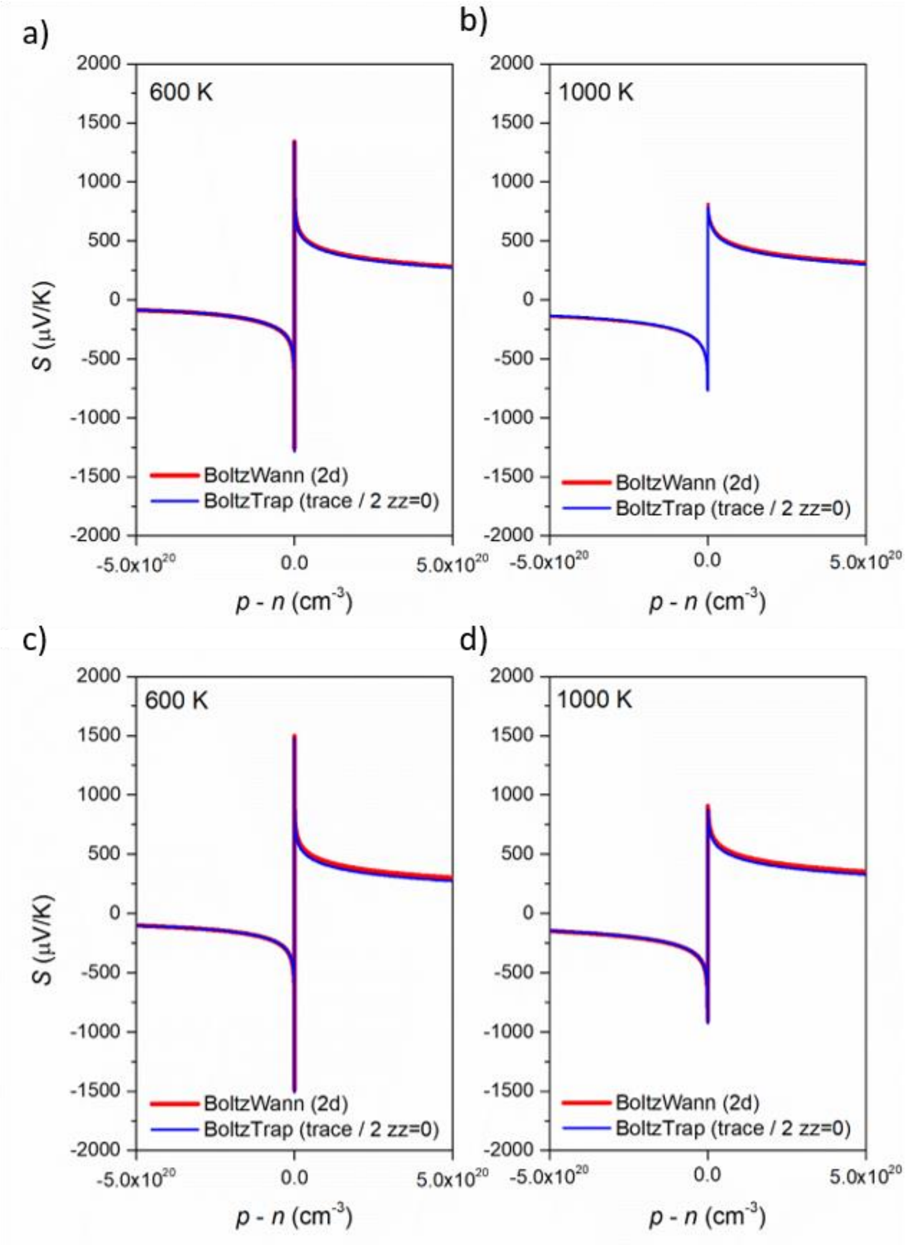

Figure S7. Comparison of the Seebeck coefficient as a function of carrier concentration for the $SrTiO_3$ (a/b) and La-doped $SrTiO_3$ (c/d) surface slab models obtained using the BoltzTraP and BoltzWann codes at T = 600 (a/c) and 1000 K (b/d).

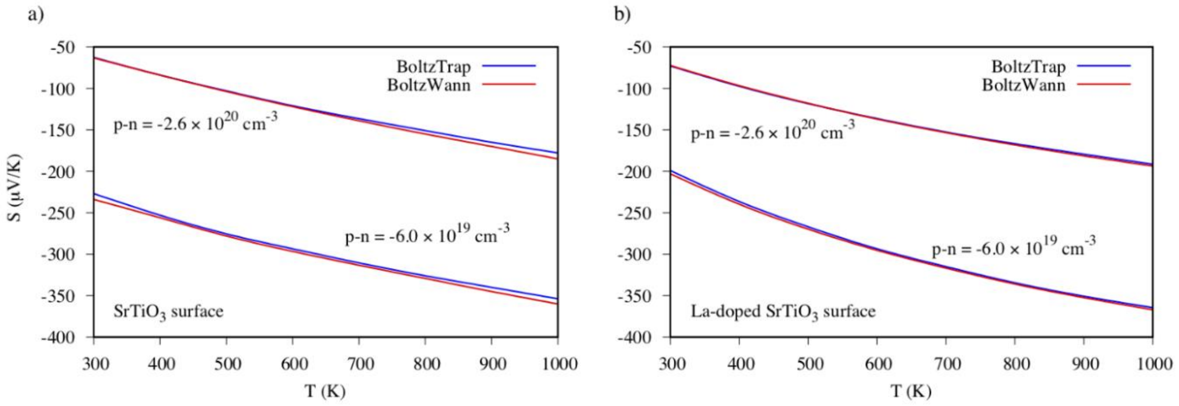

Figure S8. Temperature dependence of the Seebeck coefficients of the $SrTiO_3$ (a) and La-doped $SrTiO_3$ (b) surface model at a fixed carrier concentration of -2.6 × 10^{20} cm^{-3} (i.e. *n*-type doping), obtained using the BoltzTraP and BoltzWann codes.

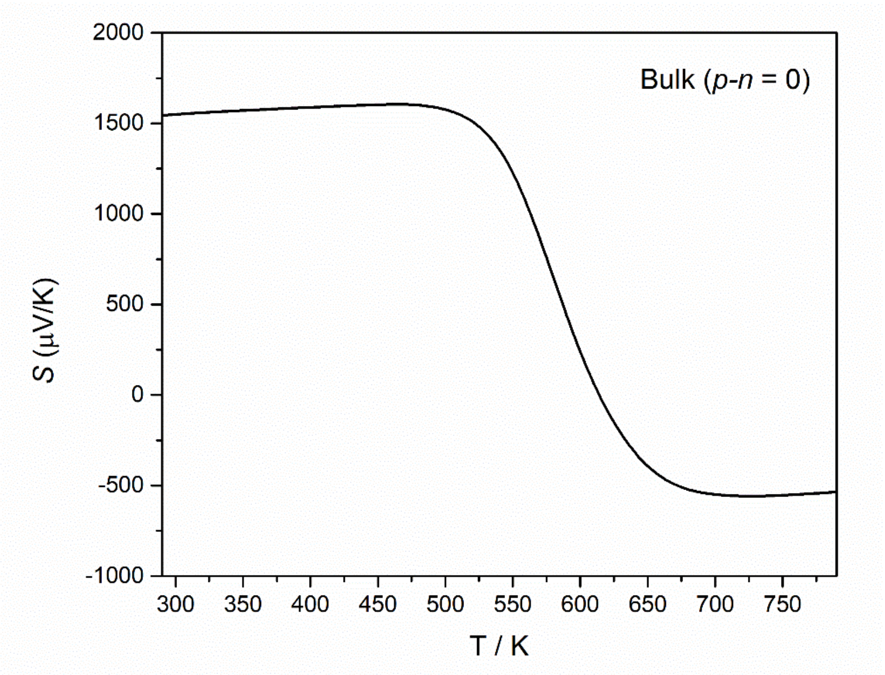

Figure S9. Temperature dependence of the Seebeck coefficient of bulk $SrTiO_3$ obtained using BoltzTraP at a carrier concentration *p*-*n* = 0.

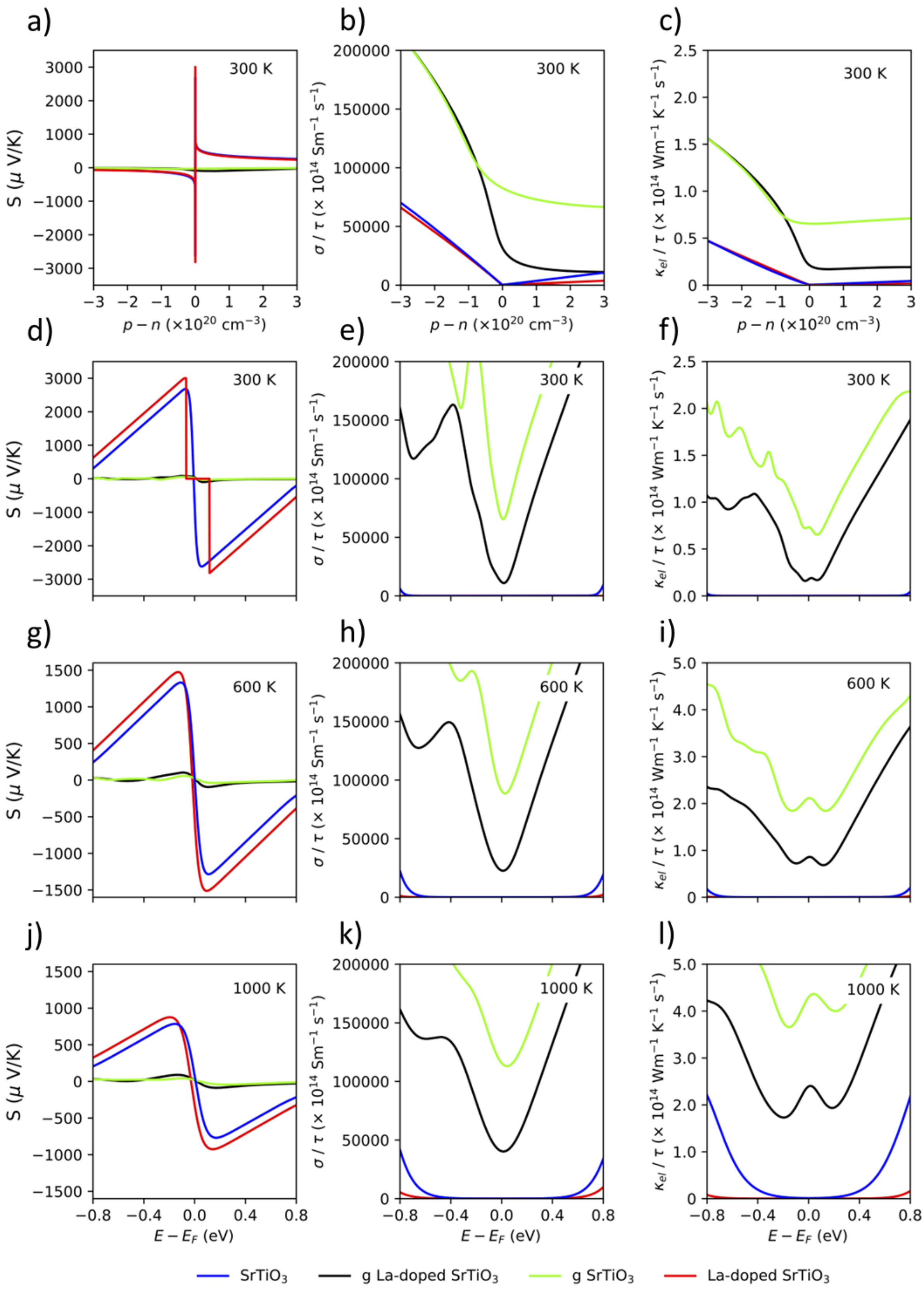

Figure S10. (a-c) Calculated Seebeck coefficient S (a), electrical conductivity σ/τ (b) and electronic thermal conductivity $\kappa_{\mathrm{el}}/\tau$ (c) as a function of carrier concentration for pristine and graphene-adsorbed $SrTiO_3$ and La-doped $SrTiO_3$ surfaces at T = 300 K, obtained using the BoltzTraP code. (d-l) Calculated Seebeck coefficient S (d/g/j), electrical conductivity σ/τ (e/h/k) and electronic thermal conductivity $\kappa_{\mathrm{el}}/\tau$ (f/i/l) as a function of chemical potential for pristine and graphene-adsorbed $SrTiO_3$ and La-doped $SrTiO_3$ surfaces at T = 300, 600 and 1000 K, obtained using the BoltzTraP code.

Rotation of Surfaces and Group Velocities

The surface-slab models employed in this work are rotated with respect to bulk $SrTiO_3$ (Figure S11). The thermal conductivity and related tensor quantities computed for the rotated cell, denoted κ_{ij}^{m}, can be related to the original cell, κ_{ij},[11] as outlined below.

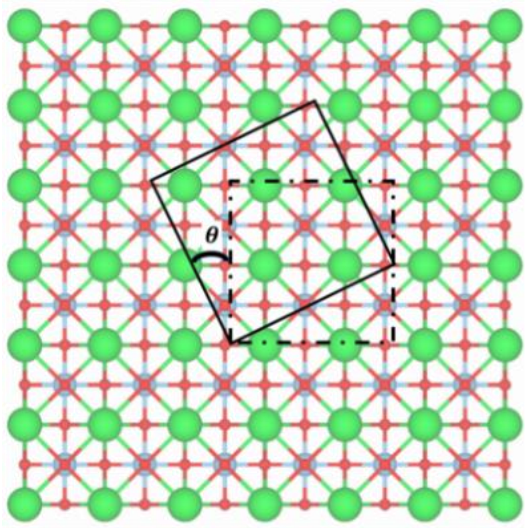

Figure S11. Rotation of the surface unit cell depicted for the $SrTiO_3$ surface lab model, where the angle ϑ = 26 **°**. The solid line marks the rotated surface cell and the dashed line the original surface cell, which corresponds to a 2 × 2 expansion of the {100} surface.

For our surface κ_{ij}^{m} is given by:

$$\kappa_{ij}^{m} = \frac{J\kappa_{ij}J^{\mathrm{T}}}{\det(J)} \qquad \text{Eq. S1}$$

$$\begin{pmatrix} \kappa_{xx}^{m} & \kappa_{xy}^{m} & 0 \\ \kappa_{yx}^{m} & \kappa_{yy}^{m} & 0 \\ 0 & 0 & \kappa_{zz}^{m} \end{pmatrix} = \begin{pmatrix} \kappa_{xx}\cos^2\theta + \kappa_{yy}\sin^2\theta & (-\kappa_{xx} + \kappa_{yy})\sin\theta\cos\theta & 0 \\ (-\kappa_{xx} + \kappa_{yy})\sin\theta\cos\theta & \kappa_{yy}\cos^2\theta + \kappa_{xx}\sin^2\theta & 0 \\ 0 & 0 & \kappa_{zz} \end{pmatrix} \qquad \text{Eq. S2}$$

where J is the Jacobian matrix of the coordinate transformation between the rotated and original surfaces, and for rotation around the z axis perpendicular to the surface:

$$J = \begin{pmatrix} \cos\theta & \sin\theta & 0 \\ -\sin\theta & \cos\theta & 0 \\ 0 & 0 & 1 \end{pmatrix} \qquad \text{Eq. S3}$$

We can solve the simultaneous equations in Eq. S2 to obtain expressions for the tensor elements for the rotated surface in terms of those of the original surface to obtain κ_{xx}, κ_{yy}, and the off-diagonal term $\kappa_{xy} = 0$:

$$\kappa_{xx}^{m} = \kappa_{xx}\cos^2\theta + \kappa_{yy}\sin^2\theta \qquad \text{Eq. S4}$$

$$\kappa_{yy}^m = \kappa_{yy}\cos^2\theta + \kappa_{xx}\sin^2\theta \quad \text{Eq. S5}$$

$$\kappa_{xy}^m = \left(-\kappa_{xx} + \kappa_{yy}\right) sin\theta cos\theta \quad \text{Eq. S6}$$

As $\cos^2\theta + \sin^2\theta = 1$, we can substitute $\cos^2\theta = 1 - \sin^2\theta$:

$$\kappa_{xx}^m = \kappa_{xx}(1 - \sin^2\theta) + \kappa_{yy}\sin^2\theta \quad \text{Eq. S7}$$

$$\kappa_{yy}^m = \kappa_{yy}(1 - \sin^2\theta) + \kappa_{xx}\sin^2\theta \quad \text{Eq. S8}$$

$$\kappa_{xy}^m = -\kappa_x \sin\theta\cos\theta + \kappa_y \sin\theta\cos\theta \quad \text{Eq. S9}$$

We now proceed to rearrange Eq. S7 so that:

$$\kappa_{xx}^m = \kappa_{xx} - \kappa_{xx}\sin^2\theta + \kappa_{yy}\sin^2\theta \quad \text{Eq. S10}$$

$$\kappa_{yy}\sin^2\theta = \kappa_{xx}^m - \kappa_{xx} + \kappa_{xx}\sin^2\theta \quad \text{Eq. S11}$$

We similarly rearrange Eq. S8 to obtain:

$$\kappa_{yy}^m = \kappa_{yy} - \kappa_{yy}\sin^2\theta + \kappa_{xx}\sin^2\theta \quad \text{Eq. S12}$$

$$\kappa_{xx}\sin^2\theta = \kappa_{yy}^m - \kappa_{yy} + \kappa_{yy}\sin^2\theta \quad \text{Eq. S13}$$

Substituting Eq. S13 into Eq. S10 gives:

$$\kappa_{xx}^m = \kappa_{xx} - \left(\kappa_{yy}^m - \kappa_{yy} + \kappa_{yy}\sin^2\theta\right) + \kappa_{yy}\sin^2\theta$$

$$= \kappa_{xx} - \kappa_{yy}^m + \kappa_{yy} - \kappa_{yy}\sin^2\theta + \kappa_{yy}\sin^2\theta$$

$$= \kappa_{xx} - \kappa_{yy}^m + \kappa_{yy} \quad \text{Eq. S14}$$

Substituting Eq. S11 into Eq. S12 gives:

$$\kappa_{yy}^m = \kappa_{yy} - (\kappa_{xx}^m - \kappa_{xx} + \kappa_{xx}\sin^2\theta) + \kappa_{yy}\sin^2\theta$$

$$= \kappa_{yy} - \kappa_{xx}^m + \kappa_{xx} - \kappa_{xx}\sin^2\theta + \kappa_{yy}\sin^2\theta$$

$$= \kappa_{yy} - \kappa_{xx}^m + \kappa_{xx} \quad \text{Eq. S15}$$

Dividing Eq. S9 by $\sin\theta\cos\theta$ gives the following relations:

$$\kappa_{yy} = \kappa_{xx} + \frac{\kappa_{xy}^m}{\sin\theta\cos\theta} \quad \text{Eq. S16}$$

$$\kappa_{xx} = \kappa_{yy} - \frac{\kappa_{xy}^m}{\sin\theta\cos\theta} \quad \text{Eq. S17}$$

Substituting Eq. S16 in to Eq. S14 yields:

$$\kappa_{xx}^{m} = \kappa_{xx} - \kappa_{yy}^{m} + \kappa_{xx} + \frac{\kappa_{xy}^{m}}{\sin\theta\cos\theta} \qquad \text{Eq. S18}$$

$$\kappa_{xx} = \frac{\kappa_{xx}^{m}}{2} + \frac{\kappa_{yy}^{m}}{2} - \frac{\kappa_{xy}^{m}}{2(\sin\theta\cos\theta)} \qquad \text{Eq. S19}$$

Similarly, substituting Eq. S17 into Eq. S15 gives:

$$\kappa_{yy}^{m} = \kappa_{yy} - \kappa_{xx}^{m} + \kappa_{yy} - \frac{\kappa_{xy}^{m}}{\sin\theta\cos\theta} \qquad \text{Eq. S20}$$

$$\kappa_{yy} = \frac{\kappa_{xx}^{m}}{2} + \frac{\kappa_{yy}^{m}}{2} + \frac{\kappa_{xy}^{m}}{2(\sin\theta\cos\theta)} \qquad \text{Eq. S21}$$

These relationships can be applied to both the thermal conductivity and the constant relaxation-time approximation function defined in Eq. 16 in the text to obtain the *xx* and *yy* components in terms of the bulk $SrTiO_3$ axes, and thence the in-plane average.

Convergence of $\boldsymbol{\kappa}_{\text{latt}}/\boldsymbol{\tau}^{\text{CRTA}}$ with respect to the q-point sampling mesh

As described in the text, we computed for each of the four surface-slab models the function $\boldsymbol{\kappa}_{\text{latt}}/\tau^{\text{CRTA}}$ defined in Eq. 16. As depicted in Figure S12, we found that the convergence of this function with respect to the **q**-point sampling mesh was somewhat erratic. To handle this, we calculated the function over a large number of meshes with systematically increasing numbers of subdivisions. We then calculated the mean value at each temperature, over all the mesh sizes tested, and removed outliers for which the calculation was more than a standard deviation from the mean. Finally, we then recalculated the standard deviation and took this as a measure of the spread, which is shown in Figure 6 in the text.

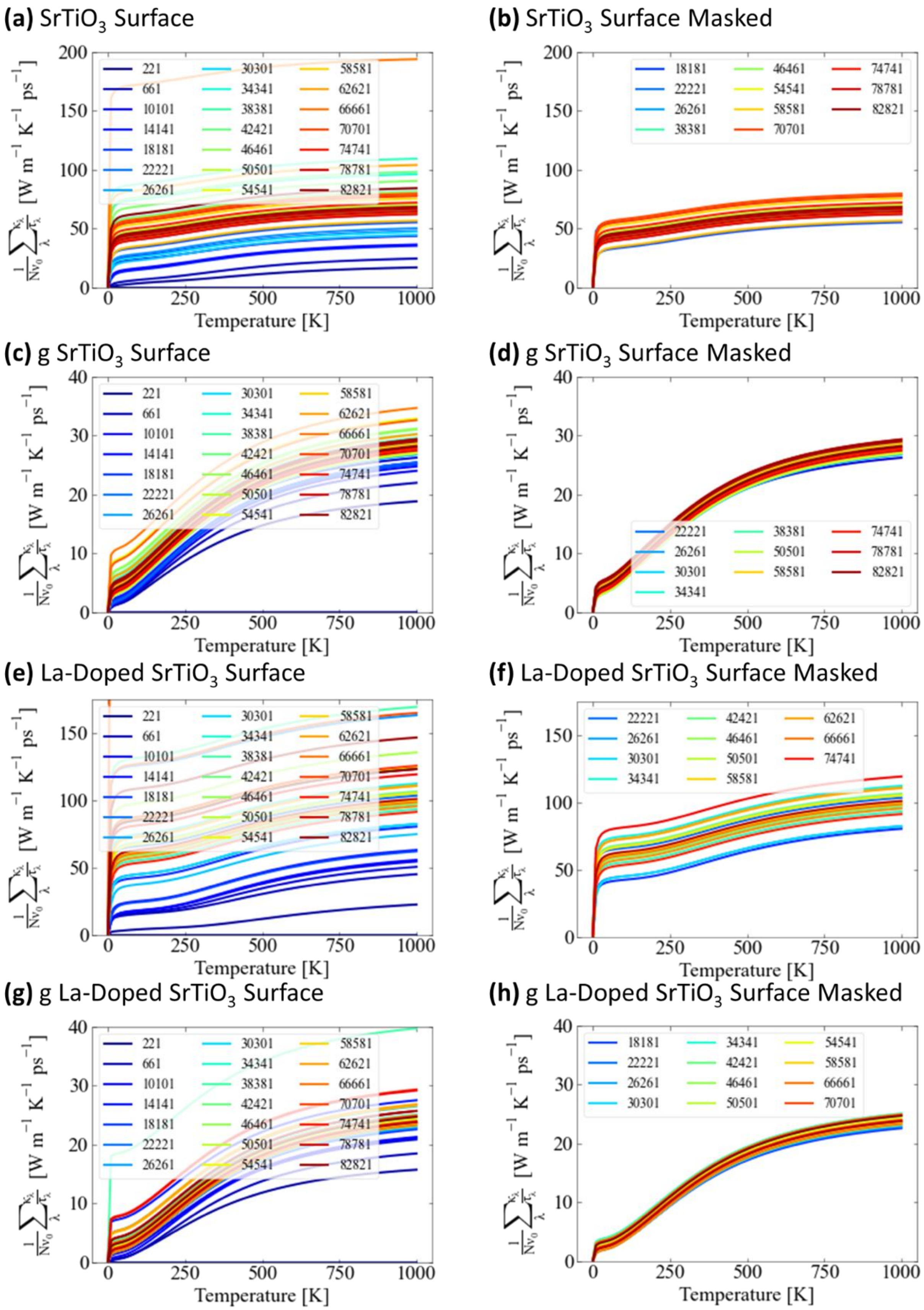

Figure S12. Convergence of the function $\boldsymbol{\kappa}_{\mathrm{latt}}/\tau^{\mathbf{CRTA}}$ (in-plane average) as a function of the **q**-point sampling mesh for the (a, b) $SrTiO_3$, (c, d) graphene-adsorbed $SrTiO_3$, (e, f) La-doped $SrTiO_3$ and (g, h) graphene-adsprbed La-doped $SrTiO_3$ surfaces. Plots in the left-hand column show the functions calculated for all the mesh sizes tested (a, c, e, g), while those in the right-hand column show the functions obtained after "masking" to remove the outliers as described above (b, d, f, h).

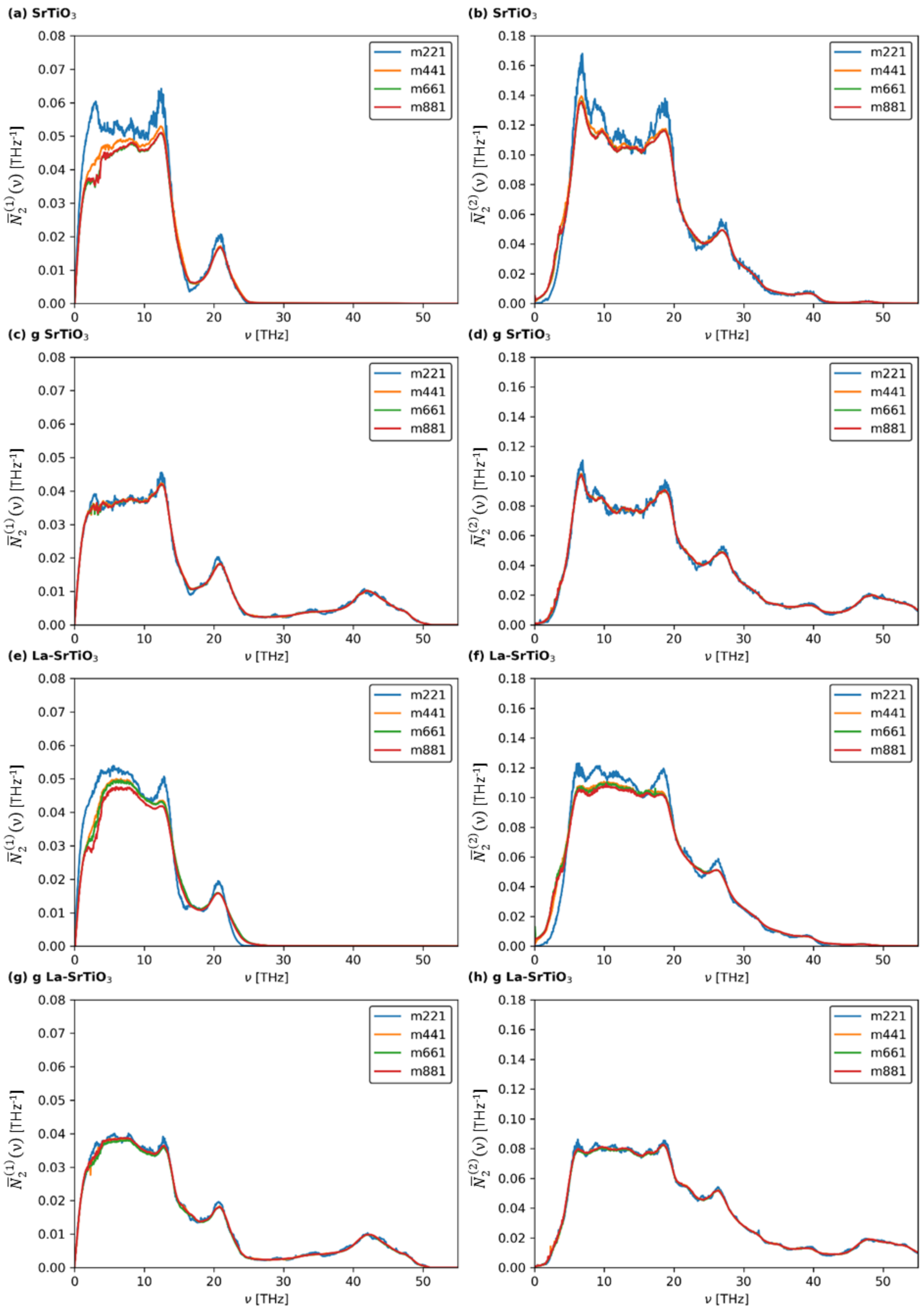

Figure S13. Convergence of the function $\overline{N}_2$ as a function of the q-point sampling mesh for the (a, b) $SrTiO_3$, (c, d) graphene-adsorbed $SrTiO_3$, (e, f) La-Doped $SrTiO_3$ and (g, h) graphene-adsorbed La-doped $SrTiO_3$ surfaces. Plots in the left-hand column show the $\overline{N}_2^{(1)}$ functions calculated for collision processes (a, c, e, g), while those in the right-hand column show the $\overline{N}_2^{(2)}$ functions calculated for decay processes (b, d, f, h).

References

(1) Pizzi, G.; Volja, D.; Kozinsky, B.; Fornari, M.; Marzari, N. BoltzWann: A Code for the Evaluation of Thermoelectric and Electronic Transport Properties with a Maximally-Localized Wannier Functions Basis. *Comput. Phys. Commun.* **2014**, *185* (1), 422–429.

(2) Pizzi, G.; Volja, D.; Kozinsky, B.; Fornari, M.; Marzari, N. An Updated Version of BOLTZWANN: A Code for the Evaluation of Thermoelectric and Electronic Transport Properties with a Maximally-Localized Wannier Functions Basis. *Comput. Phys. Commun.* **2014**, *185* (8), 2311–2312.

(3) Mostofi, A. A.; Yates, J. R.; Pizzi, G.; Lee, Y.-S.; Souza, I.; Vanderbilt, D.; Marzari, N. An Updated Version of Wannier90: A Tool for Obtaining Maximally-Localised Wannier Functions. *Comput. Phys. Commun.* **2014**, *185* (8), 2309–2310.

(4) Souza, I.; Marzari, N.; Vanderbilt, D. Maximally Localized Wannier Functions for Entangled Energy Bands. *Phys. Rev. B - Condens. Matter Mater. Phys.* **2002**, *65* (3), 1–13.

(5) Pizzi, G.; Volja, D.; Kozinsky, B.; Fornari, M.; Marzari, N. BoltzWann: A Code for the Evaluation of Thermoelectric and Electronic Transport Properties with a Maximally-Localized Wannier Functions Basis. *Comput. Phys. Commun.* **2014**, *185* (1), 422–429.

(6) Momma, K.; Izumi, F. VESTA: A Three-Dimensional Visualization System for Electronic and Structural Analysis. *J. Appl. Crystallogr.* **2008**, *41* (3), 653–658.

(7) Okuda, T.; Nakanishi, K.; Miyasaka, S.; Tokura, Y. Large Thermoelectric Response of Metallic Perovskites: $Sr_{1-x}La_xTiO_3$ (0 <~ x <~ 0.1). *Phys. Rev. B - Condens. Matter Mater. Phys.* **2001**, *63* (11), 113104.

(8) Zhang, R.-Z.; Wang, C.-L.; Li, J.-C.; Su, W.-B.; Zhang, J.-L.; Zhao, M.-L.; Liu, J.; Zhang, Y.-F.; Mei, L.-M. Determining Seebeck Coefficient of Heavily Doped La:$SrTiO_3$ from Density Functional Calculations. *Solid State Sci.* **2010**, *12* (7), 1168–1172.

(9) Ohta, S.; Nomura, T.; Ohta, H.; Koumoto, K. High-Temperature Carrier Transport and Thermoelectric Properties of Heavily La- Or Nb-Doped $SrTiO_3$ Single Crystals. *J. Appl. Phys.* **2005**, *97* (3), 34106.

(10) Ozdogan, K.; Upadhyay Kahaly, M.; Sarath Kumar, S. R.; Alshareef, H. N.; Schwingenschlögl, U. Enhanced Carrier Density in Nb-Doped $SrTiO_3$ Thermoelectrics. *J. Appl. Phys.* **2012**, *111* (5), 054313.

(11) Vemuri, K. P.; Bandaru, P. R. Geometrical Considerations in the Control and Manipulation of Conductive Heat Flux in Multilayered Thermal Metamaterials. *Appl. Phys. Lett.* **2013**, *103* (13), 133111.
